# Control of Pre-mRNA Splicing by the General Splicing Factors PUF60 and U2AF^65^

**DOI:** 10.1371/journal.pone.0000538

**Published:** 2007-06-20

**Authors:** Michelle L. Hastings, Eric Allemand, Dominik M. Duelli, Michael P. Myers, Adrian R. Krainer

**Affiliations:** Cold Spring Harbor Laboratory, Cold Spring Harbor, New York, United States of America; Centre de Regulació Genòmica, Spain

## Abstract

Pre-mRNA splicing is a crucial step in gene expression, and accurate recognition of splice sites is an essential part of this process. Splice sites with weak matches to the consensus sequences are common, though it is not clear how such sites are efficiently utilized. Using an *in vitro* splicing-complementation approach, we identified PUF60 as a factor that promotes splicing of an intron with a weak 3′ splice-site. PUF60 has homology to U2AF^65^, a general splicing factor that facilitates 3′ splice-site recognition at the early stages of spliceosome assembly. We demonstrate that PUF60 can functionally substitute for U2AF^65^ *in vitro*, but splicing is strongly stimulated by the presence of both proteins. Reduction of either PUF60 or U2AF^65^ in cells alters the splicing pattern of endogenous transcripts, consistent with the idea that regulation of PUF60 and U2AF^65^ levels can dictate alternative splicing patterns. Our results indicate that recognition of 3′ splice sites involves different U2AF-like molecules, and that modulation of these general splicing factors can have profound effects on splicing.

## Introduction

Accurate pre-mRNA splicing is essential for proper gene expression. Introns must be spliced out of pre-mRNA and exons ligated in order to make mature mRNA. Disease-causing mutations that affect the splicing process are common, and testify to the importance of splicing for normal cellular function. The splicing process is made more complex by the fact that many pre-mRNAs can be spliced in more than one way to give mature transcripts coding for proteins with distinct functions. Such alternative splicing greatly expands the coding capacity of the human genome and contributes to the overall complexity of gene expression [1]. Alternative splicing is often regulated in a tissue- and developmentally-specific manner, and is also affected by signaling pathways.

Intron removal is carried out by the spliceosome, a large complex comprised of protein and RNA components. Among these components are the U1, U2, U4, U5 and U6 small ribonucleoprotein particles (snRNPs) which consist of a specific small nuclear RNA (snRNA) and associated proteins. Assembly of the spliceosome onto pre-mRNA is a dynamic process that involves recognition of splice-site sequences at the ends of introns (reviewed in [2]). For most introns, the conserved splice-site sequences include a 5′ splice-site element surrounding a GT dinucleotide, and a 3′ splice-site AG dinucleotide preceded by a polypyrimidine tract and an upstream branchpoint sequence. These splice-site elements are initially recognized by U1 snRNP, which binds at the 5′ splice site, and by SF1/mBBP, U2AF^65^ and U2AF^35^, which bind cooperatively to the branchpoint sequence [3], [4], polypyrimidine tract [5], [6] and 3′ splice-site AG dinucleotide [5], [7], [8], [9], respectively.

A conserved family of splicing factors called SR proteins also facilitates the earliest recognition of the 5′ and 3′ splice sites (for review [2]). These first interactions between the spliceosome and the pre-mRNA are important in identifying splice sites and committing an intron to splicing. Once an intron has been initially identified, U2 snRNP becomes stably associated with the pre-mRNA. Recruitment of the U4/U6.U5 tri-snRNP to the transcript initiates the formation of a mature spliceosome that is poised to catalyze intron excision (for review, see [10]).

As a general rule, strong matches to the splice-site consensus sequences are good predictors of efficient splicing. However, there are many cases of introns with weak splice sites that are constitutively spliced, and examples of alternative splicing in which apparently weak splice sites are utlilzed more efficiently than splice sites with stronger matches to the consensus sequence [11], [12]. Our current understanding of the determinants of exon identity and intron splicing is limited. The well-defined consensus sequences at the 5′ and 3′ splice sites do not contain sufficient information to accurately identify bona fide splice sites [13], suggesting that additional sequence features are involved in the recruitment of the spliceosome to the correct location. Some of these features include exonic and intronic enhancer and silencer elements, which are recognized by distinct classes of RNA-binding proteins [14].

Based on biochemical purification of the spliceosome, nearly 200 proteins are estimated to be involved in splicing (for review, see [15]). However, only a fraction of these proteins currently have established roles in splicing. Identification of factors that are important for the recognition of weak splicing signals with poor matches to the splicing consensus sequences is an important goal towards understanding the mechanisms involved in splice-site selection and splicing regulation and fidelity. Such factors may play fundamental roles in tissue-specific or developmentally regulated splicing events, which often result from the use of apparently weak splice sites. In addition, studying the splicing of introns with weak splice sites may facilitate the identification of splicing factors that are important for splicing *in vivo*, but may be dispensable for detection of basal splicing of introns with strong consensus splice sites, which have traditionally been used in most mechanistic studies of splicing *in vitro*.

We were interested in identifying splicing factors required for the splicing of weak splice sites. We developed an in vitro splicing complementation assay in which splicing of a substrate with a weakened 3′ splice site is restored upon addition of a fraction of HeLa cell nuclear extract. We identified PUF60 as a protein that stimulated splicing in this assay. PUF60 was previously implicated in splicing [15], [16], though direct evidence for its role in the reaction was lacking. We now provide direct evidence that PUF60 is a splicing factor involved in 3′ splice-site recognition. We find that for some substrates, PUF60 can activate splicing in the absence of the related splicing factor U2AF^65^ and thus may function in a similar capacity. We further demonstrate that PUF60 and U2AF^65^ can function cooperatively in splicing, and that modulating their levels in cells affects specific alternative splicing events. Our results suggest the existence of a splicing regulatory pathway controlled by a class of general splicing factors that is involved in the recognition of the 3′ splice-site region.

## Results

### Identification of PUF60 as a Splicing Factor

**Figure 1:**
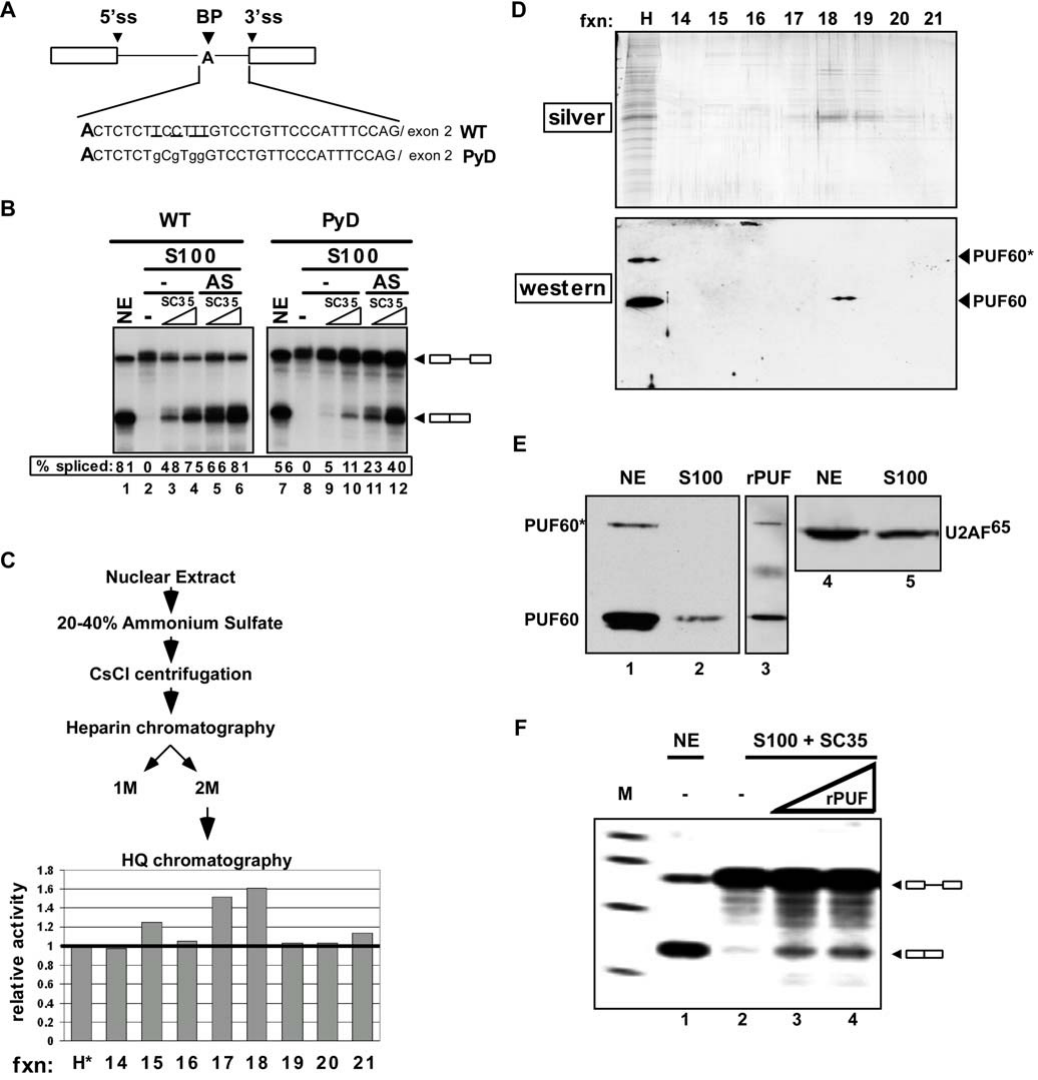
Identification of PUF60 as a Splicing Activator (A) Schematic of the wild-type (WT) and mutant (PyD) splicing substrates [19]. Boxes represent exons and lines are introns. Mutations are in lower case and the corresponding nucleotides in the wild-type substrate are underlined. Bold A indicates the branchpoint. (B) *In vitro* splicing assays were carried out in HeLa nuclear extract (NE) or S100 extract complemented with recombinant SC35, with or without a 20–40% ammonium sulfate (AS) fraction from HeLa NE. Quantitation is shown as the percent of the total RNA that is spliced. (C) Scheme for purification of the complementing activity and quantitation of RESCUE activity in HQ column fractions spanning the peak of activity. Splicing activity was normalized to the input material (H*). (see also Figure S2). H* refers to the HQ column input that was denatured with urea and renatured. (D)(top) Silver-stained SDS-PAGE of HQ peak fractions. (bottom) Western blot analysis of heparin (H) and HQ fractions 14-21. PUF* refers to an SDS-resistant dimer of the protein [16]. (E) Western blot analysis of S100 (lanes 1,4; 4 µl), NE (lanes 2,5; 4 µl), and recombinant PUF60 from E. coli (lane 3) using a PUF60 (lane 1–3) or U2AF^65^-specific (lane 4,5) antibody. (F) *In vitro* splicing assay using the PyD substrate in reactions containing S100 extract with SC35 alone (lane 2; 3 µl) and complemented with PUF60 purified from E. coli (lanes 3–4; 1 and 2 µl).

Pre-mRNA splicing *in vitro* can occur accurately in HeLa cell nuclear extract. The cytoplasmic S100 fraction obtained during the preparation of nuclear extract is also competent for splicing of many substrates when complemented with one or more SR proteins [17], [18]. We identified a pre-mRNA transcript that, as a result of mutations in the pyrimidine tract, requires additional nuclear factors to achieve high levels of splicing in S100 extract. This transcript is derived from the human β-globin gene, with modified sequences between the branchpoint sequence and the 3′ splice site (Figure 1A; [19]). In this case, the parental substrate (WT) is spliced efficiently in HeLa nuclear extract, as well as in HeLa S100 extract complemented with SR proteins, such as recombinant SC35 (Figure 1B). When four pyrimidine residues near the branchpoint sequence were substituted with guanines (PyD) (Figure 1A), there was little difference in the splicing of the two substrates in nuclear extract (Figure 1B). However, splicing of the weakened intron substrate (PyD) was severely compromised in S100 extract complemented with SC35 (Figure 1B). Deficient PyD splicing could be rescued by the addition of a 20-40% ammonium sulfate fraction of nuclear extract (Figure 1B). The ammonium sulfate fraction enhanced splicing of the WT substrate as well, though not to the same degree as it stimulated PyD. We refer to this activity that can rescue splicing of the PyD substrate as RESCUE (Required for Efficient Splicing Complementation in Unproductive Extract).

The splicing factor U2AF^65^ was a possible candidate for RESCUE activity because it recognizes the pyrimidine tract during the splicing reaction [5]. However, western analysis showed a substantial amount of U2AF^65^ in S100 extract (Figure 1E). We also found that recombinant U2AF^65/35^ that complements splicing in a U2AF depletion assay (Figure S1A) was not able to complement splicing in our S100 complementation assay (Figure S1B) indicating that U2AF^65/35^ are not responsible for RESCUE activity.

We purified RESCUE activity by sequential biochemical fractionation (Figure 1C). Following each step of purification, fractions were assayed for their activity in splicing of PyD pre-mRNA in S100 extract supplemented with SC35. Active fractions were pooled and purified further. As a first step, RESCUE activity in the 20–40% ammonium-sulfate precipitate was subjected to density-gradient centrifugation in cesium chloride (CsCl). The active fractions from the CsCl gradient were loaded on a Poros HE1 heparin column and RESCUE activity eluted at high salt (data not shown). We next disrupted protein-protein interactions in the active fractions by urea denaturation, and separated the pooled fractions on a Poros HQ column in the presence of urea (Figure 1C). Proteins associated with RESCUE activity bound to the column and were eluted at low salt concentrations (Figure S2). The most active fraction (Figure 1C, fraction 18) comprised a limited number of polypeptides, as analyzed by SDS-PAGE (Figure 1D). To identify the proteins, the entire fraction was digested with trypsin and the resulting peptides were identified by liquid chromatography tandem mass spectrometry (LC/MS/MS). Peptides from two proteins were detected: PUF60 (also known as FIR, RoBP1 and siah-bp1) and DDB1. DDB1 is a UV-damaged-DNA binding protein involved in nucleotide excision repair; it is structurally related to the U2-snRNP-associated protein SF3b130, but otherwise has no obvious link to splicing [20].

PUF60 is related to U2AF^65^, which has an important role in 3′ splice site recognition, and is thus a good candidate for RESCUE activity. We confirmed the presence of PUF60 in the HQ fraction with RESCUE activity by western blotting (Figure 1D). PUF60 migrates as a monomer and as an SDS-resistant dimer on SDS-PAGE; the latter form was detected in addition to the monomer in the heparin fraction (Figure 1D).

To confirm that PUF60 is the primary factor responsible for RESCUE activity, we generated recombinant PUF60 in E. coli (Figure 1E). The addition of rPUF60 to S100 extract with SC35 stimulated PyD splicing (Figure 1F) demonstrating that PUF60 activates PyD splicing in the RESCUE splicing assay. Western blot analysis revealed that PUF60 is present predominantly in nuclear extract, with substantially lower levels in S100 extract (Figure 1E), which explains why the addition of PUF60 to the extract stimulates splicing in our complementation assay. In contrast, the level of U2AF^65^ in S100 extract is comparable to the level in nuclear extract (Figure 1E).

PUF60 has been previously identified as a component of purified spliceosomes (reviewed in [15] and is associated with the 17*S* U2 snRNP [21]. PUF60 was originally found in a highly purified fraction of nuclear extract that contained both PUF60 and the splicing factor SRp54. This fraction, but not purified recombinant PUF60 alone, complemented splicing when combined with U2AF^65/35^ in extract depleted of poly(U)-binding proteins [16]. Because this previous report did not demonstrate that recombinant PUF60 could complement splicing, which is the definitive criterion for demonstrating a functional role for a protein, it has been unclear whether PUF60, SRp54 or additional unidentified factors were responsible for the splicing activity of the purified fraction. Thus, our finding that PUF60 can complement splicing in S100 extracts is the first formal demonstration that human PUF60 is a functional splicing factor.

### PUF60 Associates with Splicing Factors Involved in Early Spliceosome Assembly

**Figure 2:**
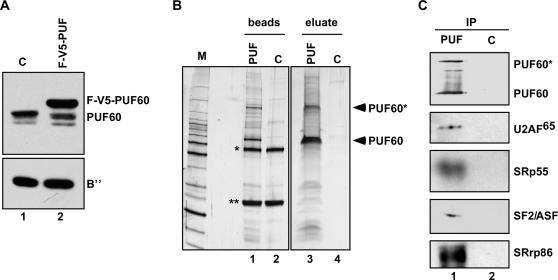
Identification of PUF60-associated Proteins. (A) Western blot analysis of nuclear extract (NE) prepared from control untransfected HeLa cells (C, lane 1) or cells expressing the FLAG-V5-tagged PUF60 protein (F-V5-PUF, lane 2). Blots were probed with antibodies specific for PUF60 and U2 snRNP B”. (B) Silver-stained 4–20% gradient SDS-PAGE of FLAG-PUF60 (PUF, lanes 1, 3) or control (C, lanes 2,4) immunoprecipitates, either bound to the α-FLAG beads (lanes 1,2) or eluted from the beads with FLAG peptide (lanes 3,4). The heavy chain (*) and light chain (**) from the FLAG antibody are indicated. (C) Western blot analysis of FLAG-PUF60 immunoprecipitates eluted with FLAG peptide.

To better understand the role of PUF60 in splicing, we identified PUF60-interacting proteins using a HeLa cell line with stable-integration of PUF60 cDNA fused to tandem N-terminal FLAG and V5 epitope tags (Figure 2A). Nuclear extract was prepared from these cells and PUF60 was immunoprecipitated with anti-FLAG antibody linked to agarose beads (Figure 2B). PUF60 and co-immunoprecipitated proteins were eluted from the beads with excess FLAG peptide, and were then separated by SDS-PAGE (Figure 2B). Prominent polypeptides were excised and identified by mass spectrometry. To thoroughly characterize proteins that specifically associate with PUF60, we performed a parallel experiment in which immunoprecipitates from F-V5-PUF60 HeLa nuclear extract or from control HeLa nuclear extract were digested with trypsin directly on the beads (Figure 2B). Released peptide fragments were subjected to analysis by mass spectrometry. Using these two methods, proteins specifically associated with PUF60 were identified. The results from both experiments were compiled, and only proteins identified in both experiments and absent from control samples were classified as PUF60-associated proteins with a high degree of confidence (Table 1).

**Table 1:** Proteins associated with PUF60

Accession #	Protein	Cal. Mass	Peptides*	log (e)*
**SR proteins**				
SW:Q05519	SRp54	53542	25	−221
SW:QWXA9	SRrp86	71649	19	−165
SW:Q07955	SF2/ASF	27745	10	−59
SW:Q13247	SRp55	39587	6	−31
SW:Q13243	SRp40	31264	4	−30
SW:Q16629	9G8	15763	5	−26
SW:Q01130	SC35	25476	3	−25
SW:Q13595	TRA-2 alpha	32689	2	−17
SW:Q15815	TRA2-beta	33666	2	−9
**U1-associated**				
SW:P08621	U1 70K	51557	4	−21
SW:P09012	U1A	31279	1	−5
**U2-associated**				
SW:O75533	SF3b155	145830	32	−239
SW:Q13435	SF3B145	97585	14	−95
SW:Q15393	SF3B130	135577	5	−39
SW:Q15459	SF3A120	88886	7	−37
SW:Q15427	SF3b50	44386	4	−37
SW:Q9Y3B4	SF3B14	14585	4	−29
SW:Q12874	SF3A60	58849	2	−17
SW:Q15393	SF3b130	135576	1	−6
SW:P09661	U2 A'	28415	1	−1.4
**Other splicing factors**				
SW:Q9UQ35	SRM300	299614	23	−171
SW:Q7L014	DDx46/prp5	117575	19	−122
SW:Q14498	HCC1	40541	7	−51
SW:095218	ZN265	36318	5	−27
SW:Q15287	RNPS1	34208	3	−23
SW:P26368	U2AF65	53501	2	−10
SW:Q9UMS4	Prp19	55180	2	−3
**RNA-related**				
SW:Q9Y383	LUC7L2	47506	8	−53
SW:P38919	eIF4AIII/DDx48	46871	6	−38
SW:Q9NQ29	LUC7L	38405	4	−21
SW:O95232	CROP(LUC7a)	51466	3	−13
SW:P35637	FUS	53426	2	−10
SW:P05455	La	46837	2	−6
SW:Q9Y580	RBM7	30503	1	−7
SW:P10155	Ro 60KDa	60670	1	−4
SW:Q14103	hnRNP D	38434	1	−2
**Others**				
NP_653205	NHN1	106378	7	−45

* Results from experiment in which proteins are directly digested and sequenced from beads.

Strikingly, most of the proteins that co-immunoprecipitated with PUF60 are known splicing factors. Nine SR proteins, as well as U1 and U2 snRNP-associated proteins make up the majority of the proteins associated with PUF60 (Table 1). These splicing factors function at early steps of the splicing reaction, during the initial recognition and specification of splicing signals. Consistent with the mass-spectrometry data, western analysis confirmed the presence of several proteins identified in the PUF60 immunoprecipitates (Figure 2C).

### Functional Redundancy and Synergy between PUF60 and U2AF^65/35^

**Figure 3:**
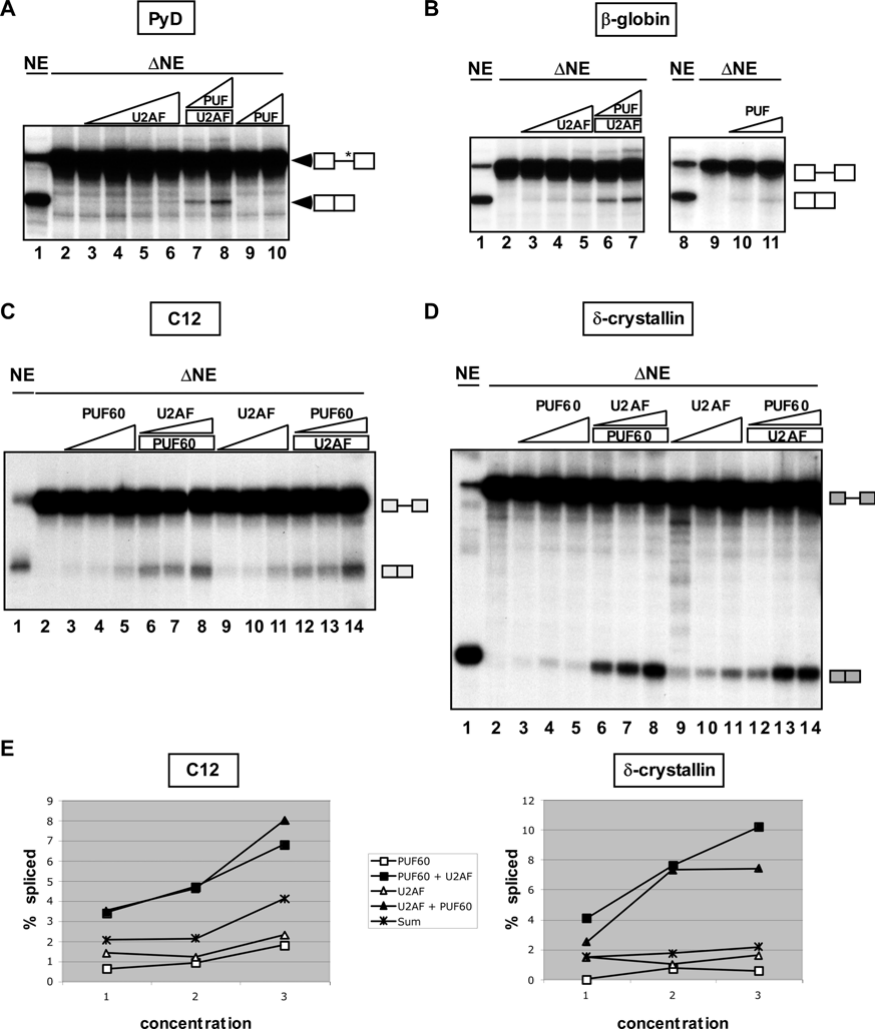
Cooperative Activity of PUF60 and U2AF^65/35^ in Splicing. (A) Complementation of PyD splicing *in vitro* in nuclear extract depleted of PUF60 and U2AF subunits using poly(U)-Sepharose. PyD pre-mRNA spliced in mock-depleted nuclear extract (NE, lane 1), extract depleted of U2AF subunits and PUF60 (ΔNE, lane 2), depleted extract complemented with U2AF^65/35^ purified from HEK-293E cells alone (lanes 3–6: 17, 33, 67, and 133 nM final concentration of U2AF^65^, respectively) or with 67 nM of U2AF^65^ plus recombinant PUF60 (lanes 7,8: 1.2 and 2.4 µM PUF60) or with PUF60 alone (lanes 9,10: 1.2 and 2.4 µM). (B) Splicing of β-globin intron 1 in nuclear extract (NE, lanes 1, 8), or in poly(U)-depleted extract alone (ΔNE, lanes 2,9). Depleted extract was complemented with purified U2AF^65/35^ (lanes 3–5: 17, 33, and 133 nM final concentration of U2AF^65^), with 670 nM of U2AF^65^ plus recombinant PUF60 (lanes 6,7: 1.2 and 2.4 µM,) or with PUF60 alone (lanes 10,11: 1.2 and 2.4 µM). (C) C12 and (D) δ-crystallin pre-mRNA spliced in nuclear extract (NE, lanes 1), depleted extract (ΔNE, lanes 2) or depleted extract with addition of recombinant PUF60 (lanes 3–5: 1.2, 2.4, and 4.8 µM final concentration; lanes 6–8: 1.2 µM; lanes 12–14, 0.6, 1.2, and 2.4 µM) or purified U2AF^65/35^ (lanes 6–8: 33, 67, and 133 nM of U2AF^65^; lanes 9–11: 67, 133, and 200 nM ; lanes 12–14: 670 nM). (E) Quantitation of C12 (left) and δ-crystallin (right) splicing. Splicing was calculated at three concentrations of protein (see C and D). PUF60 corresponds to quantitation of lanes 3–5; PUF60+U2AF^65^ refers to lanes 6–8; U2AF refers to lanes 9–11; and U2AF+PUF60 corresponds to lanes 12–14. The level of splicing expected if the PUF60 and U2AF activity is additive was calculated as the sum of lanes 3+9, 4+10, and 5+11, respectively (Sum).

PUF60 has sequence and structural homology to U2AF^65^ [22] suggesting that the two proteins may have related functions in splicing. To investigate this possibility, we compared the ability of PUF60 and U2AF^65^ to complement splicing in extract depleted of both factors. We performed poly(U)-Sepharose chromatography of nuclear extract, which effectively generates extract depleted of these proteins (Figure S3A; [16], [23]. The flow-through (NEΔ) removes more than 98% of poly(U)-binding factors, such as PUF60, U2AF^65^, and U2AF^35^ (Figure S3B, S3C and S3D) and does not support splicing of PyD or other substrates *in vitro* (Figure 3). This depletion method does not alter the levels of other nuclear splicing factors tested, such as SF2/ASF (Figure S3B).

To test the ability of U2AF and PUF60 to complement splicing in the depleted extract, we expressed His-tagged PUF60 or His-tagged U2AF^35^ in HEK-293E cells and purified the proteins by nickel-agarose chromatography (Figure S3C and S3D). His-tagged U2AF^35^ co-purifies with U2AF^65^ (Figure S3D), reflecting the strong interaction of these two proteins [16]. Purified PUF60 was unable to complement PyD splicing in the depleted extract (Figure 3A) and U2AF^65/35^ provided only very marginal complementation. However, when added in combination, PUF60 and U2AF^65/35^ stimulated PyD splicing. Our results using this depletion assay confirm our previous results from the S100-extract complementation assay (Figure 1F) that PUF60 has a role in PyD splicing, and suggest a mechanism for its activity that involves U2AF^65/35^.

We next tested whether the requirement for PUF60 in splicing and the cooperative action of PUF60 and U2AF^65/35^ are specific for PyD, or whether this activity is more general. We tested the natural β-globin intron 1, from which PyD was originally derived, and found that the purified U2AF complex activated β-globin splicing to a higher degree than PyD (Figure 3B). Similar to our results with PyD, the addition of PUF60 and U2AF^65/35^ together had a strong cooperative effect on splicing (Figure 3B). This result supports our finding that the PyD substrate is more dependent on PUF60 for splicing (Figure 1). U2AF^65^ is generally considered essential for pre-mRNA splicing [24], [25]. Surprisingly, in the absence of the U2AF heterodimer, PUF60 complemented splicing to some degree (Figure 3B). Overall, our results indicate the PUF60 and U2AF^65/35^ function synergistically in splicing, but are also able to functionally replace each other in the splicing of some, but not all substrates.

To further investigate this U2AF-independent splicing phenomenon, as well as the synergistic activity of PUF60 and U2AF^65/35^ in splicing, we tested additional substrates, including C12 [26], δ-crystallin [27] and *ftz* [28]. We found that splicing of these substrates could be complemented by PUF60 in the depleted extract (Figures 3C and 3D and S4). The complementing activities of PUF60 and U2AF^65/35^ were comparable for the C12 substrate (Figure 3C). In contrast, U2AF^65/35^ was more active than PUF60 in complementing δ-crystallin (Figure 3D) and *ftz* splicing (Figure S4). The amount of U2AF^65/35^ added to the reaction was considerably lower than that of PUF60, because the specific activity of recombinant PUF60 appears to be lower than that of U2AF^65/35^. This difference may reflect the intrinsic activities of the proteins in the splicing reaction, or it may be due to differences in the specific activities of the recombinant protein preparations. For example, the PUF60 recombinant protein may lack a cofactor—analogous to the U2AF^65/35^ relationship—that could be critical for maximum activity. In any case, our results indicate that U2AF^65/35^ is not strictly required for splicing *in vitro* when PUF60 is present.

A striking synergistic effect of PUF60 and U2AF^65/35^ on splicing was observed for all splicing substrates we tested. The addition of increasing amounts of U2AF^65/35^ to extract containing a fixed amount of PUF60, or vice-versa (Figures 3C and 3D and S4), resulted in a>5-fold stronger activation of splicing relative to reactions in which comparable amounts of PUF60 or U2AF^65/35^ were added to the extract in the absence of the other. The expected level of splicing if the effect of these proteins on splicing is additive was quantitated (Figures 3E and S4B, Sum) and was substantially lower than the observed splicing when PUF60 and U2AF^65/35^ were present together in the extract (Figures 3E and S4B). These results demonstrate the cooperative activity of PUF60 and U2AF^65/35^ in splicing, and may also suggest differences in substrate-specific requirements for these proteins.

### Cooperative Binding of PUF60 and U2AF^65/35^ to RNA

**Figure 4:**
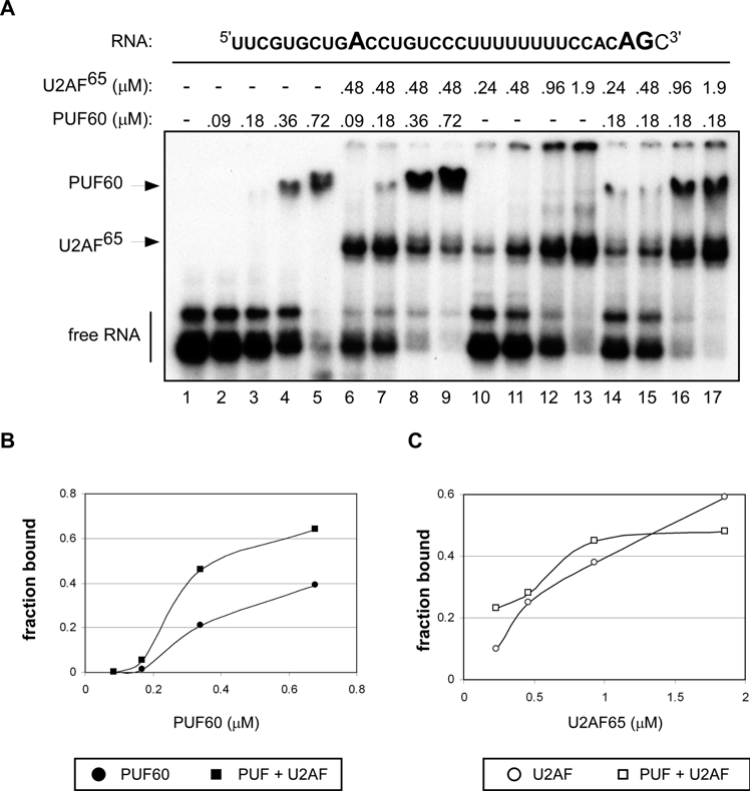
Cooperative Binding of U2AF^65/35^ and PUF60 to the 3′ splice site. (A) Electrophoretic mobility shift assay using a radiolabeled 34-nt RNA derived from adenovirus major late (AdML) pre-mRNA, and recombinant PUF60 and U2AF^65/35^. Complexes are indicated on the left. (B) Quantitation of PUF60 binding to AdML RNA represented as the fraction of total labeled RNA bound by the protein. (C) U2AF^65^ binding represented as the fraction of total labeled RNA bound by the protein.

To explore the nature of the cooperative activity of PUF60 and U2AF^65/35^, we tested whether one protein influences the binding of the other to a 3′ splice site. We performed gel-shift experiments with U2AF^65/35^ heterodimer purified from baculovirus-infected SF9 cells and recombinant PUF60 purified from human HEK-293E cells (Figure S5). A 34-nt RNA substrate derived from the 3′ end of adenovirus major late pre-mRNA (Figure 4A) was used as a binding substrate. This RNA has been extensively characterized for binding by U2AF^65/35^ and SF1/mBBP [3] and is therefore useful for assessing the general contribution of PUF60 to binding in the 3′ splice-site region. PUF60 and U2AF^65/35^ were incubated with the substrate and complexes were separated by native gel electrophoresis and detected by autoradiography and phosphorimage analysis. The proteins in the shifted complexes were identified by transferring the gels to a membrane, followed by western blot analysis using PUF60 and U2AF^65^ antibodies [29](Figure S6).

When added alone, PUF60 and the U2AF heterodimer each bound the RNA substrate (Figure 4A). When PUF60 and U2AF^65/35^ were incubated together with the RNA, PUF60 binding was enhanced as much as four-fold compared to binding in the absence of U2AF^65/35^ (Figure 4A, cf. lanes 3–5 with lanes 7–9 and lane 3 with lanes 14–17). Interestingly, increasing amounts of PUF60 binding to the RNA appeared to displace the U2AF complex, as evidenced by the decrease in the U2AF:RNA complex. Thus, U2AF^65/35^ strongly facilitates PUF60 binding and in so doing may destabilize its own binding. Alternatively, the presence of PUF60 may alter the binding of U2AF^65^ and result in a less stable interaction with the RNA that does not withstand the separation on the polyacrylamide gel. In any case, the cooperative binding of PUF60 and U2AF^65/35^ suggests that the activities of these proteins in splicing may arise from collaboration during their initial binding to the RNA.

**Figure 5:**
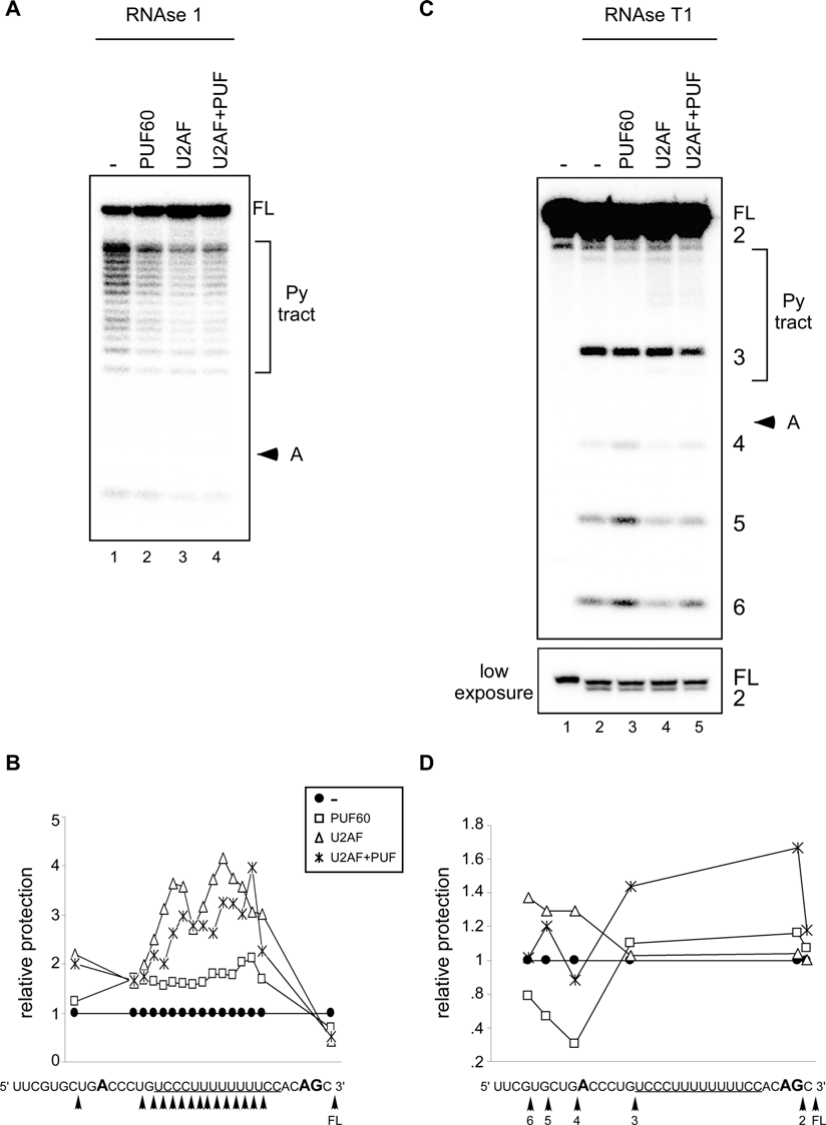
Footprinting analysis of PUF60 and U2AF^65/35^ binding (A) RNase 1 digestion. Labeled RNA was incubated with purified PUF60, U2AF^65/35^, or both PUF60 and the U2AF heterodimer, digested with RNase and separated by denaturing PAGE. The concentration of PUF60 was 0.72 µM (lane 2), or 0.36 µM (lane 4) and that of U2AF^65^ was 1.9 µM (lane 3), or 0.96 µM (lane 4). (B) Graphical representation of footprint data in (A). Relative protection is normalized to digestion in the absence of protein (A, lanes 1, 6). The RNA sequence below the plot indicates the position of cleavage (arrowhead). The pyrimidine tract is underlined. (C) RNase T1 digestion. The concentration of PUF60 was 0.36 µM (lanes 7, 9) and that of U2AF^65^ was 0.48 µM (lanes 8,9). The bands corresponding to the pyrimidine (Py) tract and branchpoint adenosine (A), and full-length RNA (FL) are indicated (see also Berglund et al., 1998). RNase T1 cleavage sites are numbered. A lower exposure of the top of the RNase T1 gel allows visualization of cleavage site 2. (D) Graphical representation of footprinting data in (C). Relative protection is normalized to digestion in the absence of protein (C, lanes 1, 6). The RNA sequence below the plot indicates the position of cleavage (arrowhead). The pyrimidine tract is underlined.

In order to help define the interaction between the 3′ splice-site region and PUF60 and the U2AF^65/35^ heterodimer and whether these interactions change when the proteins are incubated together, we performed footprinting experiments with the AdML RNA substrate and purified proteins (Figure 5). Previous footprinting analysis of this substrate with purified U2AF^65^ revealed that the protein protects the pyrimidine tract and also the branchpoint sequence to some degree [3]. To test whether both PUF60 and U2AF^65/35^ protect the pyrimidine tract, we digested the RNA with RNase 1, which cleaves 3′ of all four bases. RNase1 did not cleave efficiently near the 5′ end of the RNA, even in the absence of protein. Nonetheless, we found that the U2AF^65/35^ heterodimer and PUF60 both protected the pyrimidine tract from cleavage (Figure 5A and 5B). We did not observe changes in the protection pattern nor in the level of protection when the two proteins were added in combination (lane 4). However, in order to see efficient protection using this enzyme, a level of PUF60 and U2AF^65/35^ was required that was out of the range for cooperative interactions, as judged by the gel-shift experiments. Thus, we were not able to assess the cooperative protection of the RNA by PUF60 and U2AF^65/35^ using RNase 1.

We also performed footprinting analysis with RNase T1, which cleaves 3′ of guanosines. We observed partial protection of the guanosine at the 3′ splice-site AG dinucleotide by PUF60 and U2AF^65/35^ when incubated individually, as evidenced by the decrease in cleavage product 2 (Figure 5C and 5D). When PUF60 and U2AF^65/35^ were added together to the reaction, the protection pattern of the RNA was altered. An increase in protection of the AG dinucleotide (product 2) as well as the guanosine in the pyrimidine tract (product 3) suggests that the binding of the proteins changes when both are present. These results suggest that the cooperative binding seen in the gel shift assay may reflect interactions at the pyrimidine tract that in turn stabilize binding to the AG at the 3′ splice site.

We also reproducibly observed U2AF^65/35^ protection of the branchpoint sequence region in the absence of PUF60 (Figure 5C, lane 4, products 4,5,6), as previously reported [3]. PUF60 had the opposite effect on accessibility in this region, causing enhanced cleavage in this region (Figure 5C and 5D, product 4, 5, 6). These effects on the branchpoint sequence may reflect non-specific binding of the proteins. Interestingly, these effects were not observed when the proteins were added together, suggesting that the presence of both proteins increases or stabilizes specific binding.

### Modulation of Alternative Splicing by PUF60 and U2AF^65^

We have shown that PUF60 and U2AF^65/35^ function cooperatively during splicing *in vitro,* and that the absence of either protein does not eliminate splicing but severely compromises its efficiency. From these results, we reasoned that splicing activity and alternative splicing might be regulated *in vivo* by variations in the level of these two proteins. We found that the levels of PUF60 and U2AF^65^ do indeed vary between different cell-types. In particular, the levels of PUF60 and U2AF^65^ in HeLa cells are 3-5 fold higher than in the neuronal-like WERI-RB-1 cells (Figure S7). These results indicate that the levels of the proteins are not constant and may be controlled in a tissue-specific manner, which may contribute to differences in alternative splicing between different cells.

**Figure 6:**
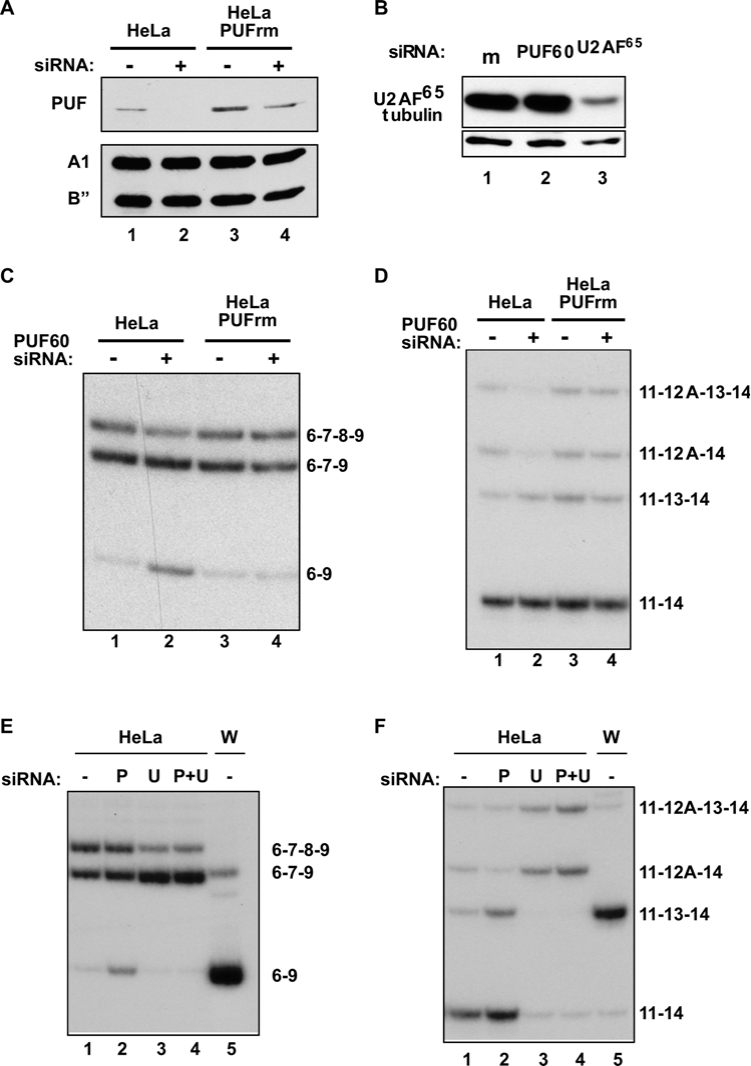
Changes in PUF60 and U2AF^65/35^ levels regulate alternative splicing in cells. (A) Western blot analysis of HeLa cells with stable expression of empty vector (HeLa) or PUF60 cDNA with silent mutations that protect transcripts from siRNA-mediated knockdown (HeLa PUFrm). Cells were treated with PUF60 siRNA (+) or mock-treated (−). Blots were probed with antibodies specific for PUF60, hnRNP A1, and U2 B”. (B) Western blot analysis of HeLa cells treated with siRNA specific for PUF60 (lane 2), a U2AF^65^-specific siRNA (lanes 3 and 4) or mock-treated (lane 1). Antibodies against U2AF^65^, PUF60, or α-tubulin were used. (C) *APP* and (D) *BIN1* alternative splicing analyzed by RT-PCR with [α-^32^P]dCTP of endogenous transcripts from stable cell lines mock-treated (lanes 1,3) or treated with PUF60 siRNA (lanes 2,4)(from (A)). (E) *APP* and (F) *BIN1* alternative splicing analyzed by RT-PCR with [α-^32^P]-dCTP of endogenous transcripts from HeLa cells mock-treated (−) or treated with PUF60 (P), U2AF^65^ (U) siRNAs, or both (P+U). RT-PCR analysis of *APP* and *BIN1* from untreated Weri-Rb1 (W) cells demonstrates neural splicing patterns.

We investigated whether alternative splicing can indeed be modulated by changing U2AF^65^ and PUF60 levels in cells. We used an RNAi approach to deplete PUF60 levels in cells. We first created a HeLa cell line (PUFrm) with stable integration of a PUF60 cDNA with silent mutations in the target region for an siRNA, and a control cell line with stable integration of the vector alone. These cell lines were treated with the PUF60 siRNA. As expected, PUF60 levels were reduced in the vector-control cell line (Figure 6A). Because PUF60rm is not targeted by the siRNA, only a slight reduction of PUF60, due to the reduction of endogenous PUF60, was seen in the PUFrm cell line (Figure 6A).

PUF60 was previously identified in a yeast three-hybrid assay as a factor that interacts with an intronic splicing enhancer located 36 nucleotides upstream of the 3′ splice-site region of the amyloid precursor protein (*APP*) transcript; this enhancer promotes inclusion of the alternatively spliced exon 8 of *APP* transcript [30]. *APP* alternative splicing is also regulated in a tissue-specific manner: neuronal tissues favor exon 7 and 8 skipping (isoform 69). whereas non-neuronal tissues exhibit nearly complete inclusion of exon 7 and some exon 8 skipping (isoforms 6789 and 679)(reviewed in [31]. To determine whether PUF60 can modulate *APP* alternative splicing, we performed RT-PCR to analyze alternative splicing of endogenous *APP* transcripts in the cells depleted of PUF60. We observed a decrease in transcripts that include exon 8, and an increase in transcripts that skip exons 7 and 8, relative to *APP* transcripts from the control cells (Figure 6C). No change in alternative splicing was observed in the knockdown of PUF60 in the PUFrm cells (Figure 6C), indicating that the change in *APP* alternative splicing was a direct and specific result of PUF60 depletion. These results are consistent with the aforementioned report that PUF60 interacts with a splicing enhancer that is important for exon 8 splicing [19]. Skipping of APP exons 7 and 8 is also the predominant isoform in WERI cells (Figure 6E), which have lower levels of PUF60 than HeLa cells (Figure S7). These findings may indicate a correlation between skipping of these exons and PUF60 levels in brain.

We next tested for changes in additional alternative splicing events following depletion of PUF60 from cells. Alternative splicing of the tumor suppressor *BIN1* is similar to that of *APP* in that there are multiple alternatively spliced exons and a distinct splicing pattern is observed in neuronally-derived samples [32]. Changes in the level of *BIN1* isoforms have been linked to tumor progression, which can be induced by modulation of the expression levels of splicing factors [32], [33]. We found that PUF60 knockdown resulted in a reduction of *BIN1* isoforms that include exon 12A (Figure 6D). This splicing pattern is similar to that observed in WERI cells (Figure 6F). Thus, knockdown of PUF60 in HeLa cells results in a shift toward a neuronal-type splicing pattern, similar to the shift in splicing observed in *APP* transcripts following PUF60 depletion.

If different introns have different requirements for PUF60 and U2AF^65^, then depleting U2AF^65^ in cells might be expected to have different effects on alternative splicing than PUF60 depletion. To test this idea, a U2AF^65^-specific siRNA was used to deplete the protein from HeLa S3 cells (Figure 6B). Consistent with above results, PUF60 depletion caused an increase in exon 7 and exon 8 skipping (Figure 6E). In contrast, U2AF^65^ depletion increased exon 8 skipping, as evidenced by an increase in isoform 679 (Figure 6E). U2AF^65^ knockdown also altered *BIN1* splicing: unlike PUF60 depletion, which favored exon 12A skipping (Figure 6F), U2AF^65^ depletion promoted exon 12A inclusion (Figure 6F). These results suggest that the splicing of different introns is differentially affected by changes in the levels of U2AF^65^ and PUF60. Knock-down of PUF60 and U2AF^65^ together resulted in a change in *APP* and *BIN1* splicing similar to the pattern seen with U2AF^65^ knockdown alone (Figure 6E and 6F), suggesting that the U2AF^65^ effect may be dominant over the PUF60 effect on splicing. We conclude from these results that modulation of the activity or levels of PUF60 and U2AF^65^ may contibute to the regulation of alternative splicing.

**Figure 7:**
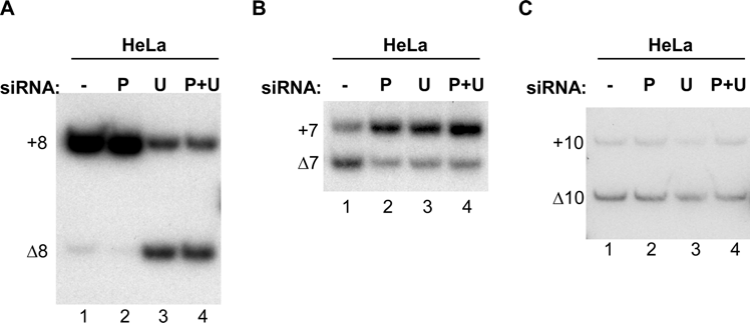
Complex modulation of alternative splicing by PUF60 and U2AF^65^. (A) *UBQLN1* exon 8 splicing, (B) *SMN2* exon 7 splicing, and (C) *MAPT* exon 10 splicing analyzed by RT-PCR in the presence of [α-^32^P]-dCTP of endogenous transcripts from untreated (−) HeLa cells, HeLa cells treated with a PUF60-specific siRNA (P), a U2AF^65^ siRNA (U), or both siRNAs (P+U). Transcripts including the alternative spliced exon (+) or skipping the exon (Δ) are labeled.

We tested the effect of PUF60 and U2AF^65^ depletion on the splicing of several additional alternatively spliced exons, and observed a spectrum of different responses. A single nucleotide polymorphism in the pyrimidine tract of *UBQLN1* intron 7 is associated with partial skipping of exon 8 and has recently been linked to an increased risk of Alzheimer's disease [34]. We found that exon 8 splicing was insensitive to changes in PUF60 levels in HeLa cells (Figure 7A). However, depletion of U2AF^65^ led to a striking increase in exon 8 skipping. We also tested splicing of *SMN2* exon 7, which is a well-studied splicing event that is influenced by a number of splicing factors (reviewed in [35]). We found that knockdown of either PUF60 or U2AF^65^ led to a decrease in exon 7 skipping (Figure 7B). Finally, we tested splicing of *MAPT* exon 10. Human mutations that alter *MAPT* exon 10 splicing are linked to frontotemporal dementia with Parkinsonism linked to Chromosome 17 (FTDP-17), and many splicing factors have been documented to be involved in the regulation of this splicing event (reviewed in [36]). We found that alternative splicing of exon 10 was not significantly affected by knockdown of PUF60 or U2AF^65^ in HeLa cells (Figure 7). Together, our results indicate that changes in the levels of PUF60 and U2AF^65^ in cells do not alter splicing of all alternative exons. Instead, changes in the quantity of these proteins appear to selectively modulate alternative splicing of a subset of exons.

## Discussion

Introns with splice-site sequences with poor matches to the consensus motifs are common in pre-mRNAs. Despite having weak splicing signals, such introns can be excised efficiently *in vivo*. The mechanisms responsible for the recognition and selection of authentic splice sites, rather than cryptic sites or alternative splicing pathways, are not clear. In particular, the highly specific recognition of 3′ splice sites is puzzling. The consensus sequence of the 3′ splice site is relatively simple, apparently requiring little more than an AG dinucleotide preceded by a region moderately enriched in pyrimidines and a degenerate branchpoint sequence. Clearly, much more is involved in defining a 3′ splice site, as this combination of sequence elements is plentiful in genomic sequences and yet 3′ splice-site selection is highly specific and subject to inactivation by single point mutations. It is apparent that our current understanding of the 3′ splice site is limited, and has likely overlooked components that may be critical for the efficient and specific recognition of sites that have poor matches to the degenerate 3′ splice-site consensus.

To gain insight into the mechanism of 3′ splice-site recognition, we investigated the factor requirements for splicing of an intron with a weak 3′ splice site/pyrimidine tract, and identified PUF60 as a critical protein for splicing (Figure 1). We showed that PUF60 stimulates splicing, and is in a complex with splicing factors involved in the early steps of spliceosome assembly (Figure 2). Mechanistically, PUF60 collaborated in a cooperative manner with U2AF^65/35^ in both RNA binding (Figures 4 and 5) and splicing activation (Figure 3). Surprisingly, however, neither protein was essential for splicing, provided that the other one was present indicating some level of functional redundancy between these structurally related proteins. In addition, modulation of the levels of U2AF^65^ and PUF60 in cells changed alternative splicing patterns (Figures 6 and 7), demonstrating that PUF60 and U2AF^65^ can modulate the efficiency of 3′ splice-site selection in a splice-site-dependent manner, and thereby regulate alternative splicing.

### PUF60: a Multi-tasking Protein

PUF60 has long been considered a putative splicing factor due to its presence in a number of purified spliceosomes (reviewed in [15]), its similarity to U2AF^65^ [22], as well as its presence in a partially purified fraction of nuclear extract with splicing activity *in vitro* [16]. In the latter study, PUF60 was shown to bind to poly(U) RNA and was the predominant protein along with SRp54 in a partially purified fraction of nuclear extract that complemented splicing of extract depleted of poly(U) binding factors. However, none of the functional assays done at that time used recombinant PUF60. Thus, despite the suggestive evidence that PUF60 was a splicing factor, rigorous proof of its function in splicing was previously lacking. To demonstrate the activity of PUF60 in splicing, we have used an S100 extract complementation assay, as well as the previous assay involving complementation of poly(U)-depleted extracts. For the latter assay, we used different substrates than Page-McCaw et al. [16], as well as recombinant PUF60 protein purified from mammalian 293 cells for our complementation; thus, it is possible that our PUF60 protein is more active and/or our splicing substrates may be more efficient or responsive to PUF60 activity.

PUF60 has other documented roles in the cell, and appears to be a protein with particularly diverse functions. PUF60 is also known as FBP-interacting repressor (FIR), a regulator of Myc gene expression [37]. In this role, PUF60/FIR represses Myc transcription in a process that involves binding between FIR and FUSE-binding protein (FBP), which binds the Myc promoter region. FIR/PUF60 itself was not found to bind the DNA, but instead enhanced FBP binding. We did not detect an association between FBP and PUF60 by western (data not shown) or by mass-spectrometry of PUF60 immunoprecipitations. However, this result does not preclude a relationship between these two proteins under other conditions. A role for PUF60 in transcription as well as splicing is intriguing, as this implies that PUF60 could contribute to the coupling between these two processes (reviewed in [38]).

Finally, PUF60 is also known as RoBPI (Ro RNA binding protein) and interacts with Ro ribonucleoproteins (RNPs) [39]. Ro RNPs have largely unknown functions, but are currently thought to play a role in quality control of small RNAs (reviewed in [40]).

### Cooperation between PUF60 and U2AF^65/35^ has a General Role in Splicing

The PyD splicing substrate with a weak pyrimidine tract was used to initially identify PUF60 as a splicing factor. Previous analysis of this substrate revealed that it requires both U2AF^65^ and U2AF^35^ for splicing, whereas splicing of the wild-type parental substrate is not dependent on U2AF^35^ ([41], Hastings&Krainer, unpublished results). U2AF^35^ recognizes the 3′ splice-site AG dinucleotide and stabilizes binding of U2AF^65^ to the pyrimidine tract [5], [7], [8], [9]. This role for U2AF^35^ may be particularly important in substrates with weak pyrimidine tracts that are not bound efficiently by U2AF^65^. Similarly, PUF60 may be required in addition to U2AF^65^ and U2AF^35^ to facilitate splice-site recognition. In these instances, in which splicing is inefficient, the synergistic activity of these proteins may be critical for splice-site identification.

We propose that 3′ splice-site selection efficiency is dictated in part by the ability of the site to be recognized by U2AF^65/35^ and PUF60. Splicing efficiency, as well as alternative splicing patterns, could thereby be dictated by the availability, modifications, or expression levels of these proteins. One possible function of the proteins may be to displace inhibitory factors from the pyrimidine tract. Indeed it has been reported that modulation of the levels of U2AF^65^ and the inhibitory protein PTB (polypyrimidine-tract-binding protein) can influence alternative 3′ splice-site selection [42]).

### Mechanistic Considerations for PUF60 in Splicing

Cooperation between PUF60 and U2AF^65/35^ was observed for all the splicing substrates tested, suggesting that this activity is an integral part of the splicing process. Synergy between proteins in splicing may reflect cooperative binding to a functional element(s), or multiple, simultaneous interactions between the activators and other components of the splicing machinery (reviewed in [43]). Indeed, we find that having both PUF60 and U2AF^65/35^ present not only stimulates splicing *in vitro* in a cooperative manner, but also influences their binding to the 3′ splice-site region (Figures 4 and 5). Our gel shift experiments suggest that PUF60 and U2AF^65/35^ may bind sequentially, rather than simultaneously to the RNA. One possible mechanism is that U2AF^65/35^ binds initially and recruits PUF60, which subsequently or concomitantly displaces U2AF from the RNA. It is also possible that U2AF is not fully displaced, but that its interaction with the 3′ splice-site is weakened in the presence of PUF60. This change in affinity could reflect an important transition in the spliceosomal complex as splicing proceeds. Although our analysis of the PUF60 complex confirmed the presence of U2AF^65^, only two peptides were found by mass spectrometry (Table 1), suggesting that interactions between the proteins may be relatively transient.

Spliceosome assembly in the 3′ splice-site region of the intron is very dynamic. Early in the process, interactions between SF1 and the U2AF heterodimer allow for cooperative RNA binding that is important for initial branchpoint sequence recognition [3]. An interaction between SF3b155 and U2AF^65^ replaces the U2AF^65^-SF1 interaction and is important for stable U2 snRNP binding to the branchpoint sequence [44]. U2AF^65/35^ binding to the RNA also becomes destabilized during this process [45]. In our PUF60 complex (Table 1) we identified SF3b155 but not SF1. One possible scenario is that SF1 binds cooperatively with U2AF^65^, which then recruits PUF60. The arrival of PUF60 could recruit SF3b155 and initiate the replacement of U2AF-SF1 with SF3b155, as well as the stable U2 snRNP association, accompanied by destabilization of U2AF^65/35^ binding. Many alternatives can also be envisioned, including the possibility that PUF60 functionally overlaps with SF1 in the recruitment of U2AF^65^ to the RNA. Such a mechanism could explain why SF1 does not appear to be essential for splicing in cells [46]. More detailed experiments aimed at understanding the mechanistic interplay of PUF60 and U2AF^65^ are required to better define the interactions of these proteins and the precise role of PUF60 in 3′ splice site selection.

The isolated PUF60 complex (Figure 2 and Table 1) offers some clues to the role of PUF60 in splicing. This complex is composed mainly of splicing factors with functions in early spliceosome assembly, including SR proteins and U1 and U2 snRNP components, as well as a putative human homolog of PRP5, an RNA-dependent ATPase. Interestingly, yeast PRP5 forms a bridge between U1 and U2 snRNPs during pre-spliceosome assembly, an association that appears to be important for U2 snRNP interaction with the pre-mRNA [47]. The presence of these particular components in the PUF60 complex further suggests that PUF60 is involved in early spliceosome assembly, perhaps by helping to recruit or stabilize U2 snRNP binding. Collectively, our results suggest that PUF60 associates with a subset of splicing factors that likely reflect its function in splicing during early events of the reaction.

### PUF60 and U2AF^65^ as a Functional Class of Splicing Factors

One model for the mechanism of PUF60 in splicing supported by our results is that PUF60 and U2AF^65^ have distinct functions in splicing, but these functions may be partially interchangeable or conditionally dispensable. Although it has been generally accepted that U2AF^65^ is required for pre-mRNA splicing in metazoans ([23] and reviewed in [22]), we demonstrate that splicing *in vitro* can occur in the absence of U2AF^65/35^ (Figure 3). Under these conditions, PUF60 is required in the extract to sustain splicing. At the same time, these two proteins act cooperatively to stimulate splicing at a level more than 5-fold greater than expected if the activities of PUF60 and U2AF^65^ were independent of each other. Thus, although splicing can occur in the absence of either protein, it is much more efficient when both are present.

Splicing was previously shown to occur in the absence of U2AF^65^ under certain experimental conditions. One report provides evidence that when nuclear extract is prepared from cells infected with adenovirus, *in vitro* splicing of some substrates is dependent on the presence of U2AF^65^ [48]; however, splicing of other substrates can occur in the absence of U2AF^65^. Another study suggesting the dispensability of U2AF^65^ reported that *in vitro* splicing can be restored in U2AF-depleted extract by the addition of an excess of the SR protein SC35 [49].

Our results raise the possibility that PUF60 and U2AF^65^ may belong to a family of factors that can modulate splicing based on substrate-specific, early recognition of distinct 3′ splice sites. Another protein, HCC1, which is structurally related to PUF60 and U2AF^65^ [50] may be another factor involved in this mode of regulation. HCC1 has been shown to interact with splicing factors such as SRp54 [51] and SRrp53 [52] and is found in the spliceosome (reviewed in [15]). Related to the notion that these proteins may represent a class of regulatory factors, a recent RNAi screen in *Drosophila* aimed at identifying splicing regulators found that knockout of *hfp*, the PUF60 ortholog, influences alternative splicing of a partially overlapping set of substrates, compared to knockout of HCC1 and U2AF50, the U2AF^65^ ortholog [53].

### Regulation of Alternative Splicing by PUF60 and U2AF^65^

If PUF60 and U2AF^65^ can indeed modulate splicing based on differential splice-site strengths and/or different requirements for their activities in the splicing of particular introns, then regulation of individual pathways via control of PUF60 and U2AF^65^ expression levels, localization, or activities could play an important role in alternative splicing and tissue-specific splicing. Indeed, we have identified several alternative splicing events that are altered by such fluctuations in cells (Figures 6 and 7).

Our observation that PUF60 depletion from HeLa cells shifts *APP* and *BIN1* processing to favor brain-specific splicing (Figure 6) suggests that PUF60 may be one factor that helps determine non-neuronal splicing patterns, and the relatively low levels of PUF60 in neuronal cell lines may be partially responsible for the observed skipping of exon exons 7 and 8 in these cells. More extensive experiments testing the effect of PUF60 over-expression in neuronally-derived cells are required to confirm this activity. In this first documented role of PUF60 in alternative splicing, the protein appears to influence splicing of some regulated exons. Interestingly, U2AF^65^ had different effects on *APP* and *BIN1* splicing compared to PUF60.

The regulation of splicing by PUF60 and U2AF^65^ appears to be complex, and at this point not readily predictable. We have identified splicing events that are only altered by U2AF^65^, others that are altered in a similar fashion by both proteins, and still other transcripts that are apparently unaffected by the depletion of either protein. There are no obvious sequence patterns in the 3′ splice sites of these transcripts that correlate with PUF60 or U2AF^65^ sensitivity. Identifying such features will be an important goal in understanding the mechanism of regulation by these splicing factors.

For some transcripts, such as *BIN1* and *SMN2*, the depletion of U2AF^65^ (*BIN1*) or both U2AF^65^ and PUF60 (*SMN2*) results in an increase in exon inclusion. These results argue that as yet unknown features of a splice site dictate its dependence on one or the other protein. For example, in the case of *SMN2*, the predominant skipping of exon 7 has been attributed to the disruption of a splicing enhancer in exon 7 [54]. This splicing enhancer is intact in the *SMN1* gene—a paralog of *SMN2*—whose transcripts efficiently include exon 7. Exonic splicing enhancers recruit U2AF^65^ to upstream 3′ splice sites [55], [56]. Thus, *SMN2* exon 7 skipping may be a direct consequence of inefficient U2AF^65^ binding. It is possible that the depletion of U2AF^65^ weakens the recognition of the exon 8 3′ splice site, but has little effect on exon 7 splicing, which is already compromised in its ability to recruit U2AF^65^. Thus, the strength of the exon 7 and exon 8 3′ splice sites may be equalized by U2AF^65^ or PUF60 depletion, and thus these sites become more competitive for pairing with the 5′ splice site of exon 6. The outcome of this shift in splice-site recognition would predict an increase in exon 7 inclusion, as observed in Figure 7. Indeed, masking the exon 8 3′ splice site with an antisense oligonucleotide results in more efficient exon 7 inclusion [57].

Further evidence of a role of PUF60 in alternative splicing *in vivo* comes from hypomorphic mutants of the *Drosophila* ortholog of PUF60, Half pint (Hfp), which exhibit alterations in developmentally regulated alternative splicing [58]. Knockout of Hfp [58] or the PUF60 ortholog in *C. elegans* [59] is embryonic lethal, indicating an essential role for the protein in invertebrate development.

### Models for Splicing Regulation by PUF60 and U2AF^65^

The knockdown of PUF60 and U2AF^65^ in cells results in changes in certain alternative splicing patterns. In cells in which PUF60 and/or U2AF^65^ levels become limiting, two possible scenarios can be envisioned for the mechanism of splicing regulation. First, the two proteins may substitute for each other in the splicing reaction, similar to our observations *in vitro*. This could mean that one can take over the function of the other, or that the activity of one can compensate for loss of the activity of other. In either case, recognition of individual splice sites may be affected differentially by the loss of one or the other protein, depending on the relative strength of a splice site's interaction with, or dependence on, PUF60 or U2AF^65^. This model involving differential dependence of individual 3′ splice sites on PUF60 and U2AF^65^ predicts an alteration in splicing patterns when one of the proteins becomes limiting. Alternatively, the lower levels of PUF60 and U2AF^65^ may result in a limited number of fully functional spliceosomes. Under such limiting conditions, stronger splice sites are predicted to out-compete weaker ones for binding by splicing factors, and thereby alter splicing patterns. Differential recognition may be based on the strength of interaction of the binding sites with splicing components, or perhaps on the presence of specific sequences that recruit PUF60 or U2AF^65^ to the intron. Overall, our results suggest that 3′ splice-site strength may be defined in part by the relative dependence on the cooperativity between PUF60 and U2AF for recognition.

## Materials and Methods

### Plasmids

To prepare pTT3-His PUFS and pTT3-HisPUFL, pGAD-GH-RoBPI-47,3 and pGAD-GH-RoBPI-144,2 (kindly provided by G. Boire, Université de Sherbrooke) were used as templates for PCR with the primers PUF60Hisstart and PUFresmutD to generate PUF60S and PUF60L (isoforms that lack or include alternative exon 5, respectively). To prepare pTT3-HisU2AF35, PET19b-U2AF35 (kindly provided by R.-M. Xu, New York University) was used as a template for PCR with the primers U2AF35HISR and U2AF35STOPL. Amplification products were digested with *Hind*III and *Bam*H I and ligated into pTT3 [60].

pGAD-GH-RoBPI-47,3 was used as a template for PCR with the primers PUF60NdeR and PUF60BamL to generate a PUFS fragment, which was digested with *Nde* I and *Bam*H I and ligated into pET9c vector (Novagen) to generate pET9c-PUF60S for expression in *E. coli*.

pMARX-PUF60rm was made by overlap-extension PCR using pGAD-GH-RoBPI-47,3 as a template and primers PUF60resmutA and PUF60resmutB and primers PUF60resmutC and PUF60resmutD. PCR-amplified products obtained with primers A and B were combined with product from reactions with primers C and D and amplified with primers A and D. Resulting DNA was digested with *Hin*d III and *Bam*H I and ligated into pTT3. This template was used as a template in PCR with primers BamPUFstartR and XhoPUFstop. The amplified product was digested with *Bam*H I and *Xho* I and ligated into pMarxIVpuro (kindly provided by Greg Hannon, Cold Spring Harbor Lab).

To construct pBabe-F-V5-PUF60, the *Bgl* II restriction site in PUF60L cDNA was mutated by overlap-extension PCR using pTT3-HisPUFL as a template and the primers forwardA and reverseA. The product was amplified by PCR using the primers forwardB and reverseB and cloned in the *Bam*H I and *Eco*R I restriction sites of a modified pBluescript vector carrying at the N-terminus the sequence coding for FLAG and V5 antigen (E. A. and A.R.K., unpublished). The resulting vector was digested with *Bgl* II-*Eco*R I and the F-V5-PUF60 fragment was subcloned into *Bam*H I-*Eco*R I-digested pBabe Puro vector.

The sequences of all primers used for PCR amplification are shown in Table S1.

Templates for *in vitro* splicing were βWT and βPyD (kindly provided by R.Reed, Harvard Medical School) linearized with BamH I and transcribed with SP6 RNA polymerase; β-globin linearized with BamH I and transcribed with SP6 polymerase [61]; C12 (kindly provided by T. Nilsen, Case Western Reserve University) linearized with Bgl II and transcribed with T3 RNA polymerase; ftz (kindly provided by R. Reed, Harvard Medical School) linearized with EcoRI and transcribed with T7 RNA polymerase, and δ-crystallin linearized with SmaI and transcribed with Sp6 RNA polymerase.

### RT-PCR

RNA was collected using Trizol Reagent (Invitrogen). Reverse transcription was performed using a First-strand cDNA synthesis kit (Amersham) with oligo dT primer. PCR with AmpliTaq Gold (Roche) was carried out for 30 amplification cycles (95°C for 30 s, 58–60°C for 60 s, and 72°C for 60 s) in reactions containing [α-^32^P]dCTP. Primers for RT-PCR are provided in Table S1. PCR analysis of *SMN2* exon 7 splicing was performed as previously described [54]. Products were separated on 6% native polyacrylamide gels. Quantitation was based on phosphorimage analysis (Fujix BAS2000 or Fujifilm FLA-5100).

### Cell fractionation, *in vitro* transcription, and splicing

Frozen HeLa cells were prepared as described [17] and resuspended in an equal volume of buffer A (10 mM Hepes-KOH pH 8, 10 mM KCl, 1.5 mM MgCl_2_, 1mM DTT, 0.5mM PMSF). Cells were lysed using a Dounce homogenizer. Nuclei were recovered and resuspended in an equal volume of buffer C (20 mM Hepes-KOH pH 8, 0.6 M KCl, 1.5 mM MgCl_2_, 0.2 mM EDTA, 25% (v/v) glycerol, 1 mM DTT, 0.5 mM PMSF) and lysed in a Dounce homogenizer, followed by rocking for 30 min at 4°C. The supernatant following centrifugation was dialyzed against buffer D (20 mM Hepes, 100 mM KCl, 0.2 mM EDTA, 0.5 mM PMSF, 1mM DTT, 20% (v/v) glycerol). This nuclear extract was diluted 3-fold with buffer E (20 mM Hepes-KOH pH 8, 0.2 mM EDTA, 1 mM DTT) and mixed with buffer E-AS (saturated with ammonium sulfate) to obtain a final concentration of 20%-saturated ammonium sulfate. The mixture was rotated for 60 min at 4°C and centrifuged. Dry ammonium sulfate (0.11 g/ml) was added to the supernatant and dissolved by rotation at 4°C for 45 min and centrifuged. The pellet was resuspended in buffer D and dialyzed into buffer D to yield the 20–40% AS fraction.

CsCl gradient centrifugation was carried out by addition of dry CsCl (1 g/ml) to the 20–40% AS fraction and handled as described [62]. Gradient fractions were dialyzed into buffer D. Active fractions were pooled and loaded onto a 1×10 cm Poros 20 Heparin column on an AKTA Purifier (Amersham Pharmacia). Bound proteins were eluted by stepwise washes of buffer D-1M NaCl, and buffer D-2M NaCl. The 2M eluate was dialyzed against buffer D, denatured by the addition of solid urea to a final concentration of 6M and loaded onto a 1×5 cm Poros 20 HQ column equilibrated in buffer D-6M urea. Proteins were eluted with a linear gradient from buffer D-0.1 M to -2 M NaCl with 6M urea. Fractions were dialyzed against buffer D-0.1 M KCl.

Fraction 18 from the Poros HQ column was digested with trypsin and peptides were analysed by liquid chromatography-MS/MS using 75-µm×15-cm C18 picofrit columns (New Objectives) coupled to an LTQ mass spectrometer and peptides were eluted using a 10–85% MeOH gradient in 0.5% acetic acid. Peptide fragmentation spectra were extracted using the READW program and searched using X!Tandem.

*In vitro* transcription and splicing reactions were carried out as described [63]. Nuclear extract were prepared as described [64]. PUF60 and U2AF^65/35^ were depleted from HeLa nuclear extract by poly(U)-Sepharose chromatography as previously described [5]. Products were separated on denaturing polyacrylamide gels. Quantitation was based on phosphorimage analysis (Fujix BAS2000 or Fujifilm FLA-5100).

### Western blot analysis

Western blotting was performed using rabbit polyclonal antibodies specific for PUF60 (kindly provided by G. Boire, Université de Sherbrooke), or U2AF^35^ (kindly provided by B. Graveley, University of Connecticut Health Center), and SRrp86 (kindly provided by J. Patton, Vanderbilt University), and mouse monoclonal antibodies specific for human U2-B” snRNP protein (mAb 4G3), PUF60 (M.L.H. and A.R.K. unpublished data), U2AF^65^ (A.R.K., unpublished), SRp55 (L. Manche and A.R.K., unpublished data), SF2/ASF (mAb96), hnRNP A1 (mAb A1/55, L. Manche and A.R.K., unpublished), V5 (Invitrogen), and α-tubulin (Sigma). Quantitation was performed using Alexafluor 532 anti-mouse or Alexafluor 488 anti-rabbit secondary antibodies (Molecular Probes) followed by analysis on a Fujifilm Fluor Imager FLA-5100.

### Tissue culture and transfection

PUF60rm cell lines were generated by retroviral transduction with pMarx-PUF60rm or vector alone as described [65]. The HeLa S3 cell line expressing F-V5-PUF60L was generated by viral infection with pBabe-F-V5-PUF60L as described [66]. A clonal HeLa S3 cell line stably expressing the tagged protein at a high level was selected by immunofluorescence using the anti-V5 antibody (Invitrogen) and expanded to prepare nuclear extract [64].

### RNA interference

10^5^ untransfected HeLa cells or HeLa cells expressing either pMarx or pMarx-PUF60rm were seeded into 6-well plates 24 h before transfection of siRNA with Oligofectamine (Invitrogen). The siRNAs used were: PUF60 r(GCAGAUGAACUCGGUGAUG)dTdT (sense strand, Dharmacon) and U2AF^65^ (U2AF2: r(GCAAGUACGGGCUUGUCAA)dTdT (sense strand, Qiagen). After 72 h cells were harvested for RNA isolation and western blotting.

### Recombinant proteins

E. coli-derived recombinant PUF60 was prepared from BL21 cells expressing pET9c-PUF60S. Cell pellets were sonicated in buffer D and, following centrifugation, MgCl_2_ (15 mM final concentration) was added to the supernatant. Proteins were precipitated on ice for 10 min and centrifuged. The pellet was resuspended in buffer D, sonicated, and treated to another round of precipitation as above. The final pellet was resuspended in buffer D and loaded on a heparin column with 100 mM NaCl. PUF60 was present in the flow-through, which was dialyzed overnight in buffer D with 5% (v/v) glycerol.

Mammalian-cell-derived PUF60 and U2AF^65/35^ were expressed in 293E cells transiently transfected with pTT3-HisPUFS or pTT3-HisU2AF35 in a procedure adapted from a published method [60]. For purification, cell pellets were resuspended in lysis buffer (50 mM Tris-HCl, pH 8, 1% NP-40, 5 mM imidazole, 5 mM NaF, 5 mM β-glycerophosphate, 1 mM DTT), sonicated and centrifuged. Supernatant was added to a 0.5 ml Ni-NTA agarose (Qiagen), and rotated at 4°C for 1 h. The slurry was packed on a column and the beads were washed with 50 mM Tris, 0.5 M NaCl, 5 mM imidazole. Bound protein was eluted with 50 mM Tris, 500 mM NaCl, 0.5 M imidazole, and dialyzed into buffer D. Protein concentrations were estimated by comparing protein preparations to serial dilutions of a bovine serum albumin (BSA) standard in SDS-PAGE gels stained with Coomassie Brilliant Blue R (Sigma). The purified U2AF heterodimer has a U2AF^35^ to U2AF^65^ stoichiometry of ∼3.5∶1.

Baculovirus-derived recombinant human SC35 and U2AF^65/35^ were purified from infected SF9 cells as described previously ([56], [63], respectively). The purified U2AF heterodimer has a U2AF^35^ to U2AF^65^ stoichiometry of ∼1∶1

### Immunoprecipitation

Nuclear extract prepared from the HeLa S3 cell line expressing F-V5-PUF60L or from standard HeLa S3 cells were dialyzed into IP buffer (20 mM Hepes, pH 8, 150 mM KCl, 1.5 mM MgCl_2_, 0.5 mM PMSF, 5% (v/v) glycerol), centrifuged to remove insoluble material, and incubated with rotation for 1 h at 4°C with ANTI-FLAG M2 Affinity Gel (Sigma) which had been washed three times with IP buffer containing 0.05% (v/v) Triton X-100. 1 ml of nuclear extract was added to 20 µl of beads. Following incubation, the beads were washed once with IP buffer except with 250 mM KCl, 0.05% (v/v) Triton X-100 and 200 ng/ml of RNase A, twice with the same buffer without RNase A, and twice with IP buffer with 100 mM KCl. Beads with bound protein were either directly digested with trypsin and analyzed by LCQ MS/MS or were eluted in IP buffer with 100 mM KCl and 100 ng/ml Flag peptide (Sigma). Eluted proteins were separated on an SDS-PAGE gradient gel, and major peptides were excised, digested with trypsin, and identified by LC-MS/MS as above.

### Gel-shift assay

Proteins were incubated with radiolabeled RNA (∼0.2 nM final concentration) in binding buffer [25 mM Tris (pH 7.5), 25 mM NaCl, 1 mM EDTA] with 0.1 mg/ml tRNA and 0.5 mg/ml BSA for 60 min at room temperature. RNA and RNA-protein complexes were separated in 0.5 TBE 6% native polyacrylamide gels run at 100V in the cold room. Binding was quantitated by calculating the fraction of bound RNA (specific protein-RNA complex) relative to all other unbound or bound RNA.

### Footprint analysis

Reactions were assembled identical to those in gel-shift assays, except that RNasin (Promega) was included at a final concentration of 1 U/ml. After a 30-min incubation, tRNA (2.6 mg/ml final concentration) and either RNase T1 (Ambion, final concentration of 0.06 U/µl) or RNase 1 (Ambion, final concentration of 0.6 U/µl) was added to the reaction and incubated for 5 min at room temperature followed by phenol extraction and ethanol precipitation. Products were separated on a 20% denaturing polyacrylamide gel.

## Supporting Information

Figure S1 Analysis of U2AF^65^ for RESCUE activity. (A) Recombinant U2AF^65/35^ complements splicing in depleted extracts. In vitro splicing assay using the β-globin WT construct in reactions containing nuclear extract (NE) or extract depleted of U2AF^65/35^ (ΔNE, lane 2), or depleted extracts with recombinant U2AF^65/35^ (lane 3) from baculovirus or U2AF^65^ from E. coli (lane 4). (B) In vitro splicing assay using the PyDsubstrate in reactions containing nuclear extract (lane 1) S100 extract alone (lane 2) or with SR proteins (lane 3) or S100 extract with SR proteins and recombinant baculovirus U2AF^65/35^ (lanes 4–5), or U2AF^65^ purified from E. coli (lane 6). Unspliced pre-mRNA and spliced mRNA are indicated.
(0.83 MB TIF)

Figure S2 Purification of RESCUE activity by HQ chromatography. (A) Column profile. Fractions with RESCUE activity from the Poros 20 heparin chromatography step were loaded onto a Poros 20 HQ column in low salt under denaturing conditions, and the proteins were eluted by a salt gradient. The A280 (blue), A260 (red), and conductivity (brown) and gradient (green) tracings are shown. The peak splicing activity as detected by in vitro splicing is indicated. (B) In vitro splicing of PyD pre-mRNA. Fractions from the gradient and flow-through were assayed in reactions containing nuclear extract (NE), S100 extract (S), or S100 extract and SC35 without (-) or with gradient fractions. H refers to the active fraction from the heparin column. H* refers to the active heparin fraction after denaturation and renaturation with urea, analogous to the treatment of the HQ fractions.
(6.80 MB TIF)

Figure S3 Analysis of PUF60 and U2AF^65/35^ depletion from HeLa nuclear extract. (A) Scheme for the fractionation of nuclear extract using poly(U)-Sepharose resin. (B) Western blot analysis of fractions. Δ refers to the depleted nuclear extract (column flow-through), W refers to the 2M NaCl wash, E represents the 2M guanidinium-HCl eluate, and PUF refers to recombinant PUF60 (lane 5, ∼6 pmol). (C) Analysis of extract depletion and relative levels of recombinant PUF60 and (D) U2AF^65/35^ used for complementation in Fig. 3. Western blot analysis of serial dilution of nuclear extract (lanes 1–6) compared to depleted extract (Δ, lane 7). The PUF60 blot shows His-tagged PUF60 (∼3.6 pmol) purified from HEK-293E cells (lane 8). Approximately 60% of the protein forms an SDS-resistant dimer (*). The monomer corresponds to about 1.4 pmol/μl. Quantitation of the signals indicates that 3.4 pmol of PUF60 corresponds to ∼80% of the PUF60 in nuclear extract. The U2AF^65/35^ purified protein preparation from HEK-293E cells expressing His-tagged U2AF^35^ was analyzed by western (∼4.2 pmol U2AF^35^ and ∼1.2 pmol U2AF^65^, as estimated by comparison to bovine serum albumin standard) and compared to the standard curve for nuclear extract (lanes 1–6). The purified U2AF^65^ and U2AF^35^ from HEK-293E cells correspond to approximately 9 and 17% of the concentration of U2AF^65^ and U2AF^35^ in nuclear extract, respectively. Blots were probed with antibodies specific to the indicated protein. (E) Complementation of in vitro splicing of PyD pre-mRNA in nuclear extract depleted of PUF60 and U2AF subunits. PyD pre-mRNA spliced in nuclear extract (NE, lane 1), depleted extract with the PUF60-containing 2M NaCl wash only (lane 2), or complemented also with human recombinant U2AF^65/35^ purified from baculovirus-infected SF9 cells.
(1.32 MB TIF)

Figure S4 Cooperative activity of PUF60 and U2AF^65/35^ in ftz splicing in vitro. (A) ftz pre-mRNA spliced in nuclear extract (NE, lane 1), extract depleted of U2AF subunits and PUF60 (ΔNE, lane 2), depleted extract complemented with recombinant HEK-293E-expressed PUF60 alone (lanes 3–5: 1.2, 2.4, 4.8 μM final concentration, respectively), or PUF60 (1.2 {lower case}M final concentration) with recombinant U2AF^65/35^ purified from HEK-293E cells (lane 6–8: 33, 67, 133 nM final concentration of U2AF^65^, respectively), or with U2AF^65/35^ alone (lanes 9–11: 67, 133, 200 nM of of U2AF^65^). (B) Quantitation of ftz splicing with the three concentrations of proteins shown in (A). The level of splicing expected if the PUF60 and U2AF activity is additive was calculated as the sum of lanes 3+9, 4+10, and 5+11, respectively (Sum).
(0.82 MB TIF)

Figure S5 Recombinant PUF60 and U2AF^65/35^. Coomassie-blue-stained SDS gel of recombinant PUF60 purified from HEK-293E cells (∼0.2 μg, lane 1), and recombinant U2AF^65/35^ heterodimer purified from baculovirus-infected SF9 cells (lane 2; 0.25 and 0.12 μg, respectively). Bovine serum albumin (BSA) was included to confirm the protein concentration (lanes 3–6; 0.05, 0.1, 0.2 and 0.4 μg, respectively).
(0.21 MB TIF)

Figure S6 Shift-western blot analysis. (A) Gel-shift analysis of the ^32^ P-labeled AdML 3′ splice-site fragment incubated alone (-, lane 1) or in the presence of PUF60 (lanes 2–7, 10–13) and/or U2AF^65^ (lanes 5–12). Reactions were separated on a 6% native polyacrylamide gel and electrophoretically transferred to sandwiched nitrocellulose and nylon membranes. The nitrocellulose membrane binds the protein and the RNA is transferred to the nylon membrane which is shown. (B) Western blot analysis of nitrocellulose membranes prepared as described above using an antibody against U2AF^65^. (C) Gel-shift analysis of the ^32^ P-labeled AdML 3′ splice-site fragment incubated alone (-, lane 1) or in the presence of PUF60 (lanes 2–7, 12–15) and/or U2AF^65^ (lanes 5–15). Reactions were treated as described above and nylon membrane with immobilized RNA is shown. (D) Western blot analysis of the gel in (C) using a PUF60-specific antibody.
(5.60 MB TIF)

Figure S7 Cell-type-specific APP and BIN1 splicing and PUF60 and U2AF^65^ expression. (A) Western blot analysis of whole-cell extracts (∼2.5, 5, and 10×104 cell equivalents, lanes 1–3 and 4–6, respectively) from WERI (lane 1–3) and HeLa cells (lane 4–6) separated by 12% SDS-PAGE. Blots were probed with antibodies specific to PUF60 and α-tubulin (top) or to U2AF^65^ and α-tubulin (middle). (B) Quantitation of PUF60 and U2AF^65^ protein levels. Blots were probed with a fluorescent secondary antibody and fluorescence was quantitated on a Fujifilm FLA-5100. The measurements showed a direct linear relationship between increasing amounts of input sample and fluorescence. Error bars represent the S.E.M of the three measurements from the blot shown in A.
(0.40 MB TIF)

Table S1 Sequences of primers used in PCR reactions.
(0.05 MB DOC)
