# Supplementary figures and images for: Control of Pre-mRNA Splicing by the General Splicing Factors PUF60 and U2AF65
Source: PLoS One. 2007 Jun 20;2(6):e538. doi: 10.1371/journal.pone.0000538 (PMC1888729; doi:10.1371/journal.pone.0000538)

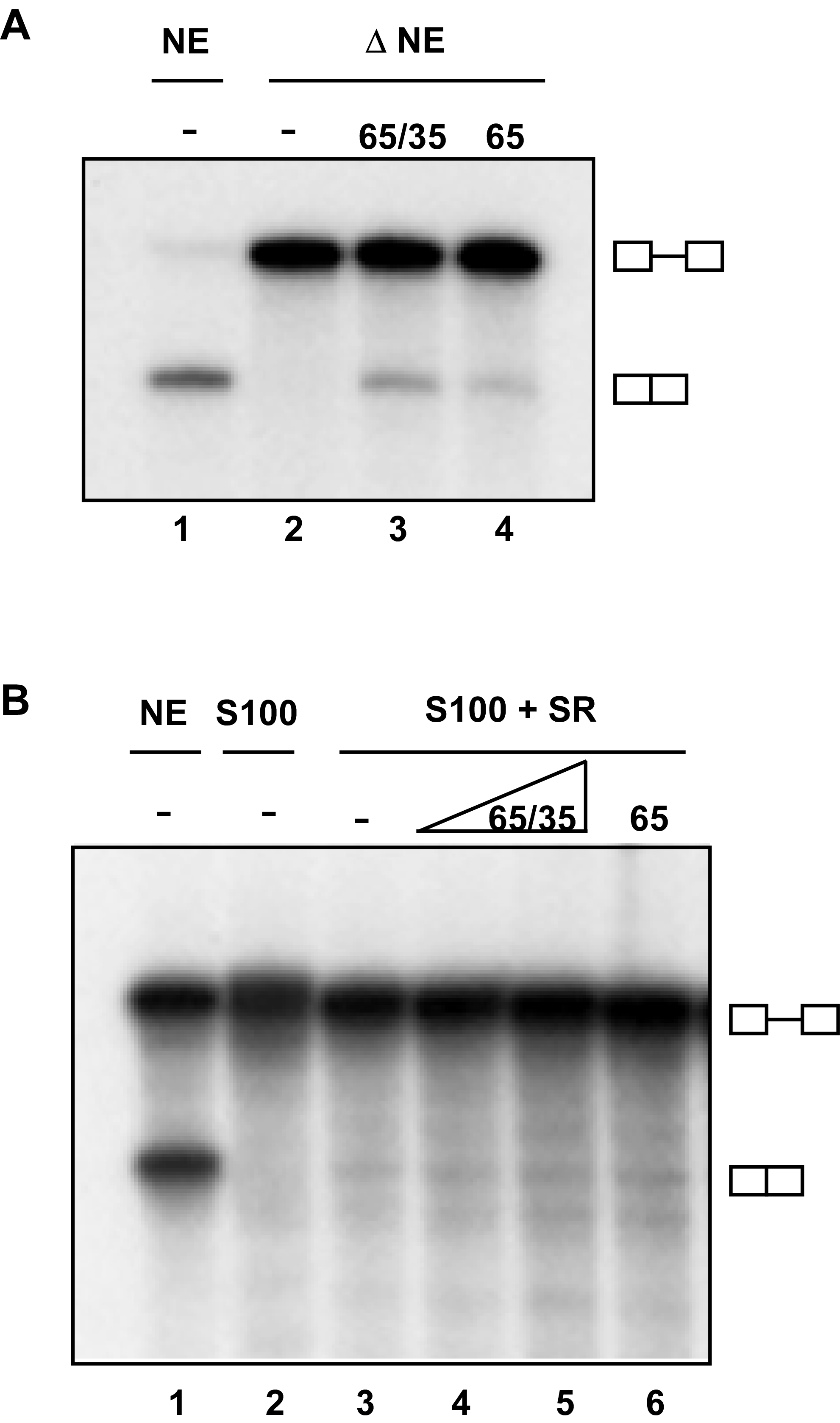

Supplement: Figure S1 — Analysis of U2AF65 for RESCUE activity. (A) Recombinant U2AF65/35 complements splicing in depleted extracts. In vitro splicing assay using the β-globin WT construct in reactions containing nuclear extract (NE) or extract depleted of U2AF65/35 (ΔNE, lane 2), or depleted extracts with recombinant U2AF65/35 (lane 3) from baculovirus or U2AF65 from E. coli (lane 4). (B) In vitro splicing assay using the PyDsubstrate in reactions containing nuclear extract (lane 1) S100 extract alone (lane 2) or with SR proteins (lane 3) or S100 extract with SR proteins and recombinant baculovirus U2AF65/35 (lanes 4–5), or U2AF65 purified from E. coli (lane 6). Unspliced pre-mRNA and spliced mRNA are indicated. (0.83 MB TIF) [file pone.0000538.s001.tif]

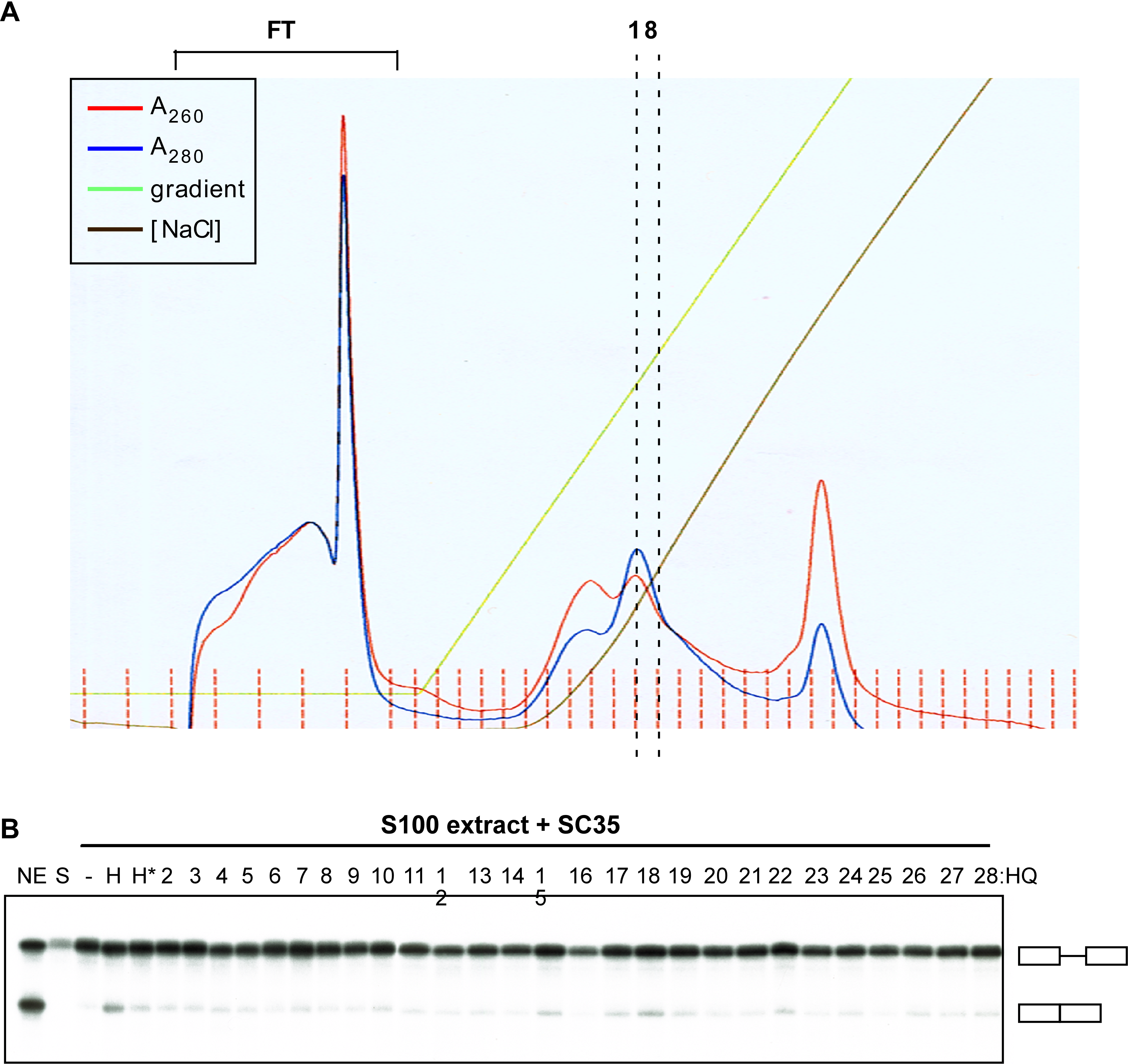

Supplement: Figure S2 — Purification of RESCUE activity by HQ chromatography. (A) Column profile. Fractions with RESCUE activity from the Poros 20 heparin chromatography step were loaded onto a Poros 20 HQ column in low salt under denaturing conditions, and the proteins were eluted by a salt gradient. The A280 (blue), A260 (red), and conductivity (brown) and gradient (green) tracings are shown. The peak splicing activity as detected by in vitro splicing is indicated. (B) In vitro splicing of PyD pre-mRNA. Fractions from the gradient and flow-through were assayed in reactions containing nuclear extract (NE), S100 extract (S), or S100 extract and SC35 without (-) or with gradient fractions. H refers to the active fraction from the heparin column. H* refers to the active heparin fraction after denaturation and renaturation with urea, analogous to the treatment of the HQ fractions. (6.80 MB TIF) [file pone.0000538.s002.tif]

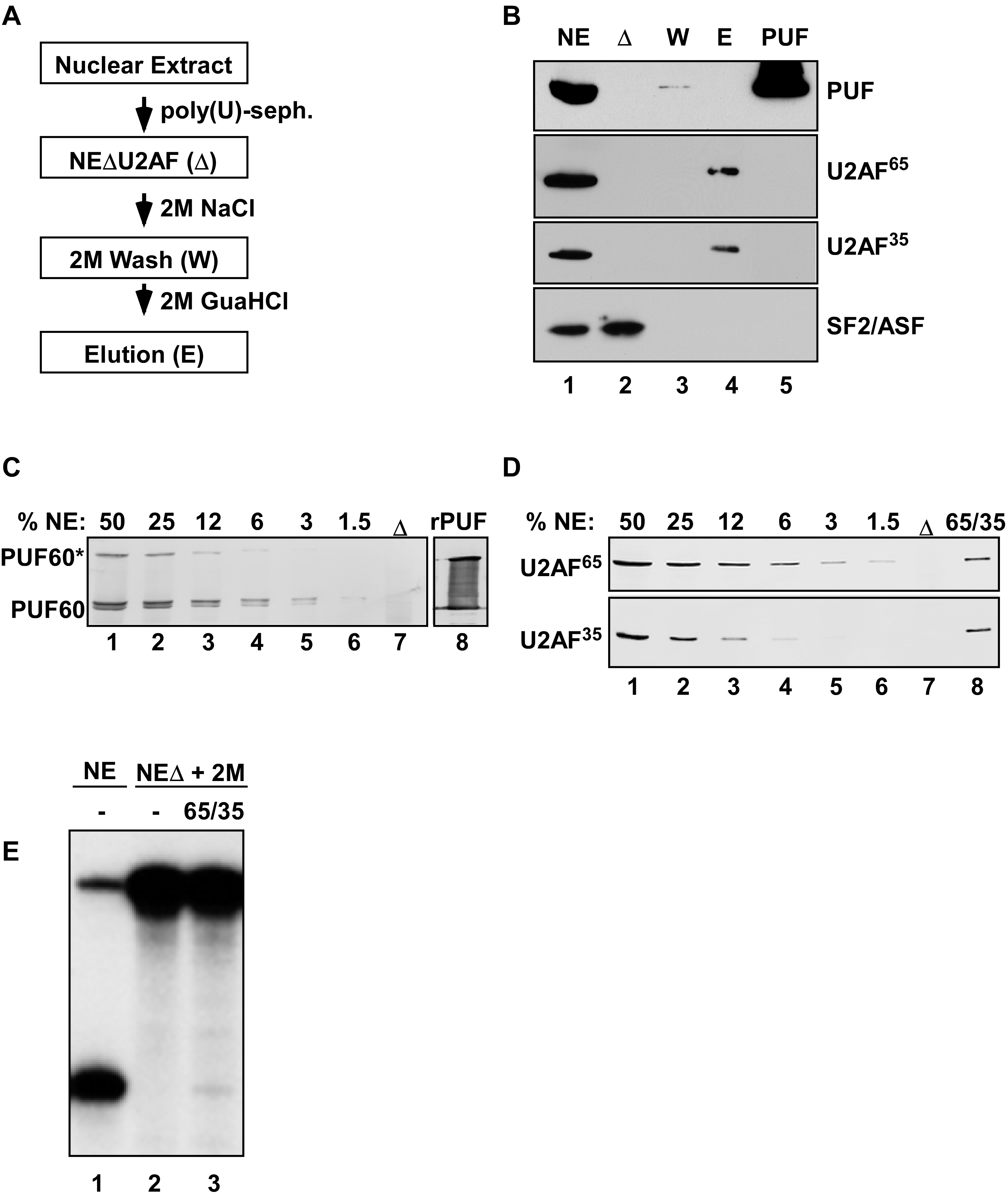

Supplement: Figure S3 — Analysis of PUF60 and U2AF65/35 depletion from HeLa nuclear extract. (A) Scheme for the fractionation of nuclear extract using poly(U)-Sepharose resin. (B) Western blot analysis of fractions. Δ refers to the depleted nuclear extract (column flow-through), W refers to the 2M NaCl wash, E represents the 2M guanidinium-HCl eluate, and PUF refers to recombinant PUF60 (lane 5, ∼6 pmol). (C) Analysis of extract depletion and relative levels of recombinant PUF60 and (D) U2AF65/35 used for complementation in Fig. 3. Western blot analysis of serial dilution of nuclear extract (lanes 1–6) compared to depleted extract (Δ, lane 7). The PUF60 blot shows His-tagged PUF60 (∼3.6 pmol) purified from HEK-293E cells (lane 8). Approximately 60% of the protein forms an SDS-resistant dimer (*). The monomer corresponds to about 1.4 pmol/μl. Quantitation of the signals indicates that 3.4 pmol of PUF60 corresponds to ∼80% of the PUF60 in nuclear extract. The U2AF65/35 purified protein preparation from HEK-293E cells expressing His-tagged U2AF35 was analyzed by western (∼4.2 pmol U2AF35 and ∼1.2 pmol U2AF65, as estimated by comparison to bovine serum albumin standard) and compared to the standard curve for nuclear extract (lanes 1–6). The purified U2AF65 and U2AF35 from HEK-293E cells correspond to approximately 9 and 17% of the concentration of U2AF65 and U2AF35 in nuclear extract, respectively. Blots were probed with antibodies specific to the indicated protein. (E) Complementation of in vitro splicing of PyD pre-mRNA in nuclear extract depleted of PUF60 and U2AF subunits. PyD pre-mRNA spliced in nuclear extract (NE, lane 1), depleted extract with the PUF60-containing 2M NaCl wash only (lane 2), or complemented also with human recombinant U2AF65/35 purified from baculovirus-infected SF9 cells. (1.32 MB TIF) [file pone.0000538.s003.tif]

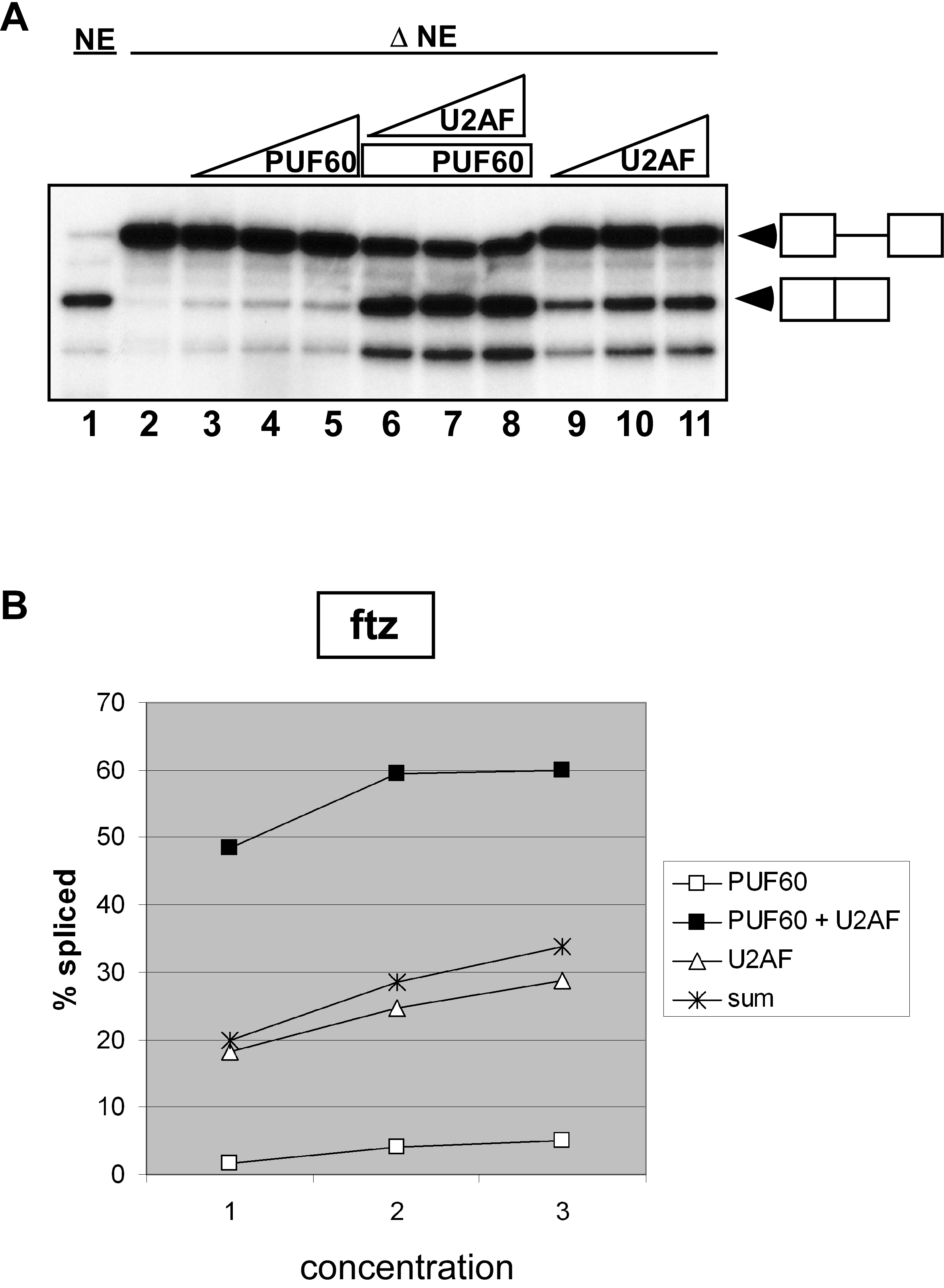

Supplement: Figure S4 — Cooperative activity of PUF60 and U2AF65/35 in ftz splicing in vitro. (A) ftz pre-mRNA spliced in nuclear extract (NE, lane 1), extract depleted of U2AF subunits and PUF60 (ΔNE, lane 2), depleted extract complemented with recombinant HEK-293E-expressed PUF60 alone (lanes 3–5: 1.2, 2.4, 4.8 μM final concentration, respectively), or PUF60 (1.2 {lower case}M final concentration) with recombinant U2AF65/35 purified from HEK-293E cells (lane 6–8: 33, 67, 133 nM final concentration of U2AF65, respectively), or with U2AF65/35 alone (lanes 9–11: 67, 133, 200 nM of of U2AF65). (B) Quantitation of ftz splicing with the three concentrations of proteins shown in (A). The level of splicing expected if the PUF60 and U2AF activity is additive was calculated as the sum of lanes 3+9, 4+10, and 5+11, respectively (Sum). (0.82 MB TIF) [file pone.0000538.s004.tif]

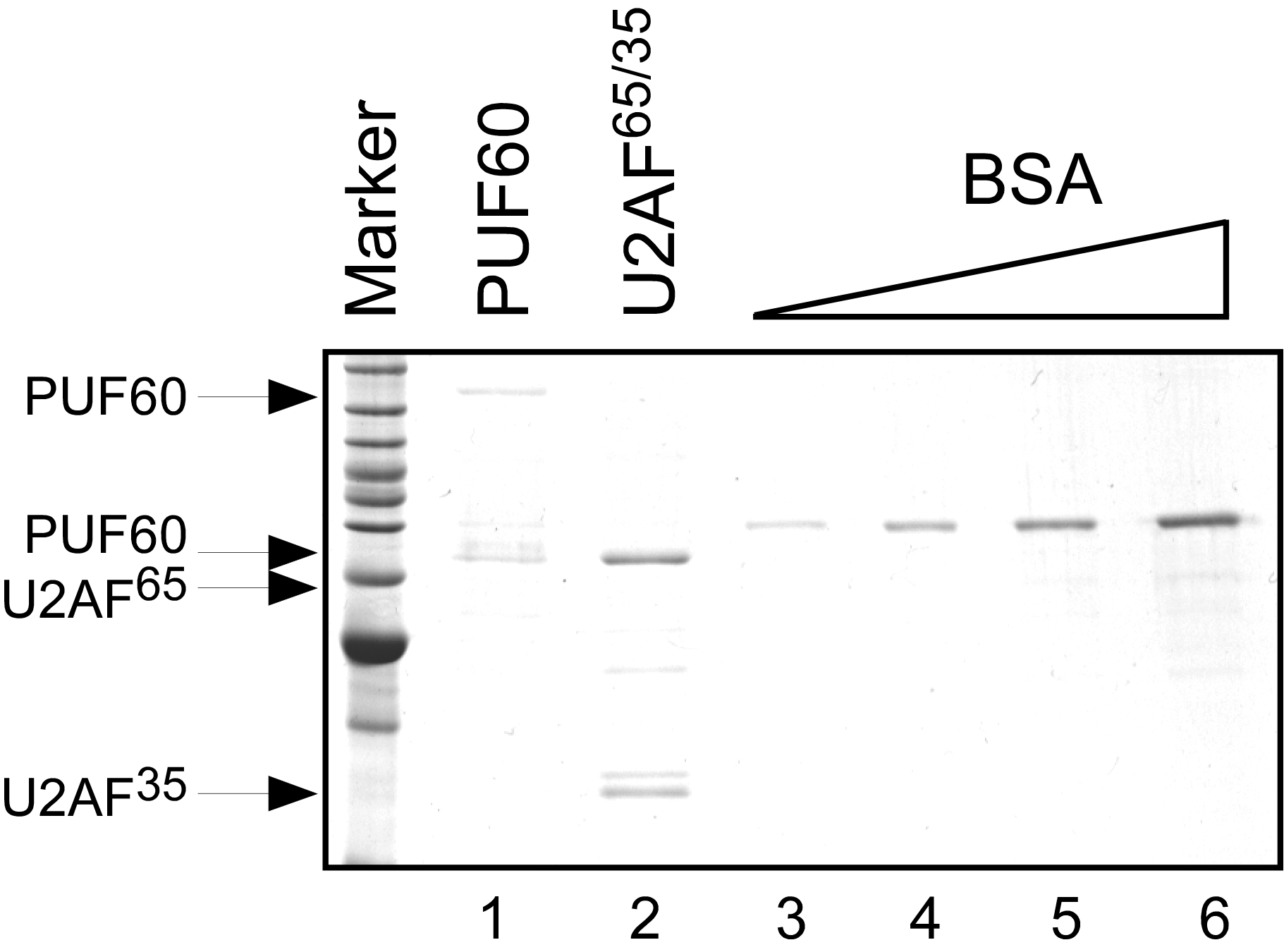

Supplement: Figure S5 — Recombinant PUF60 and U2AF65/35. Coomassie-blue-stained SDS gel of recombinant PUF60 purified from HEK-293E cells (∼0.2 μg, lane 1), and recombinant U2AF65/35 heterodimer purified from baculovirus-infected SF9 cells (lane 2; 0.25 and 0.12 μg, respectively). Bovine serum albumin (BSA) was included to confirm the protein concentration (lanes 3–6; 0.05, 0.1, 0.2 and 0.4 μg, respectively). (0.21 MB TIF) [file pone.0000538.s005.tif]

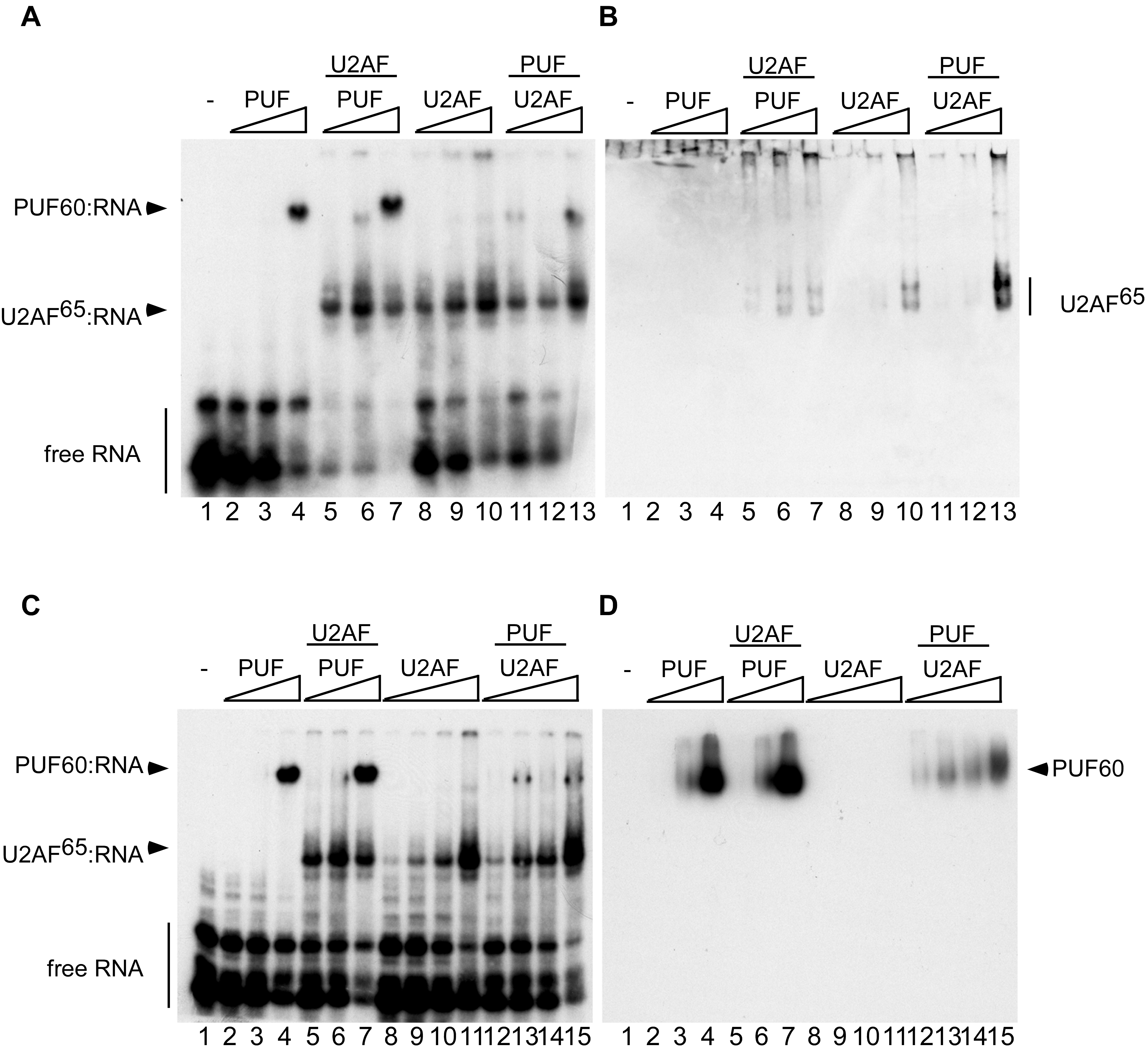

Supplement: Figure S6 — Shift-western blot analysis. (A) Gel-shift analysis of the 32 P-labeled AdML 3′ splice-site fragment incubated alone (-, lane 1) or in the presence of PUF60 (lanes 2–7, 10–13) and/or U2AF65 (lanes 5–12). Reactions were separated on a 6% native polyacrylamide gel and electrophoretically transferred to sandwiched nitrocellulose and nylon membranes. The nitrocellulose membrane binds the protein and the RNA is transferred to the nylon membrane which is shown. (B) Western blot analysis of nitrocellulose membranes prepared as described above using an antibody against U2AF65. (C) Gel-shift analysis of the 32 P-labeled AdML 3′ splice-site fragment incubated alone (-, lane 1) or in the presence of PUF60 (lanes 2–7, 12–15) and/or U2AF65 (lanes 5–15). Reactions were treated as described above and nylon membrane with immobilized RNA is shown. (D) Western blot analysis of the gel in (C) using a PUF60-specific antibody. (5.60 MB TIF) [file pone.0000538.s006.tif]

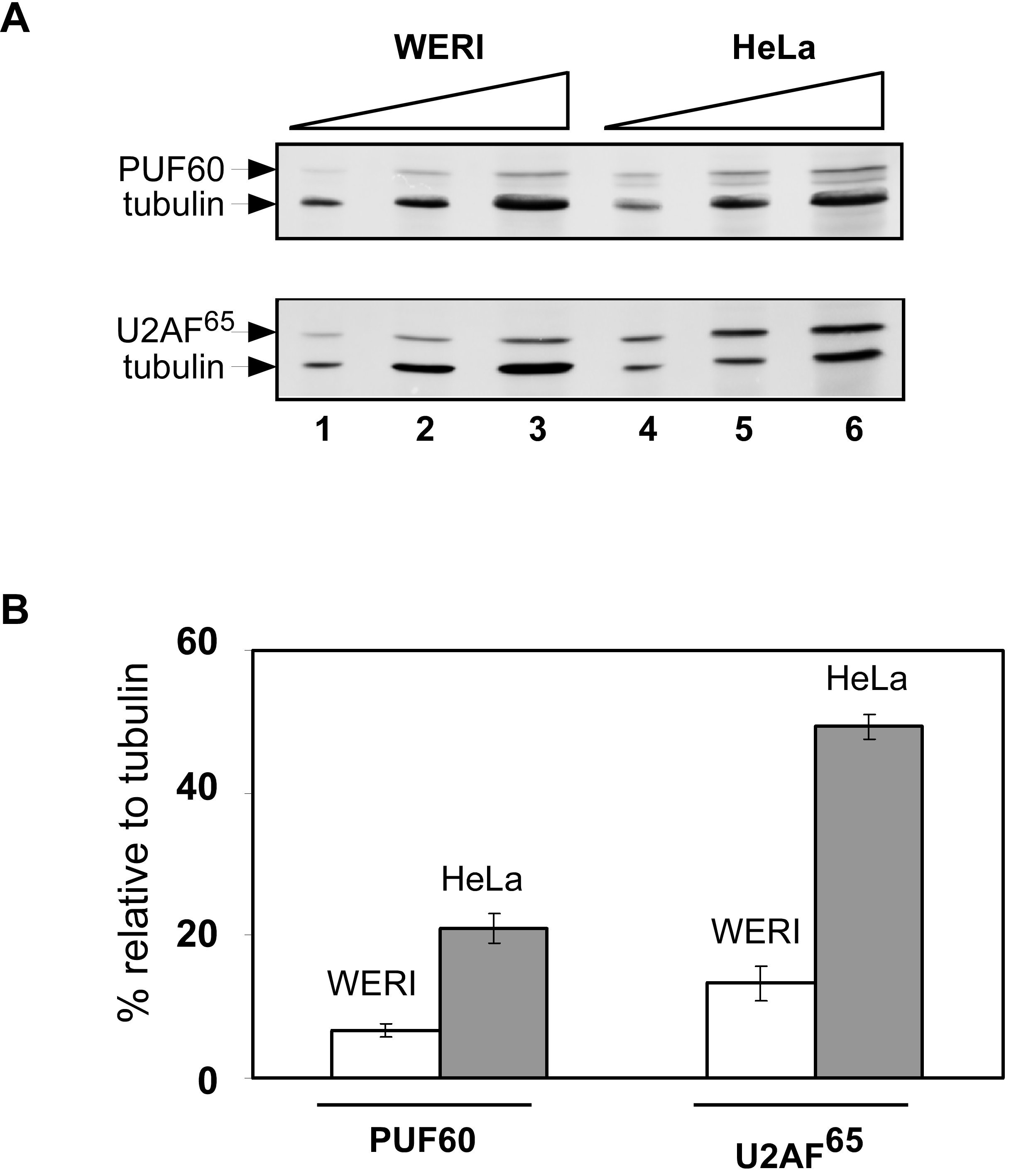

Supplement: Figure S7 — Cell-type-specific APP and BIN1 splicing and PUF60 and U2AF65 expression. (A) Western blot analysis of whole-cell extracts (∼2.5, 5, and 10×104 cell equivalents, lanes 1–3 and 4–6, respectively) from WERI (lane 1–3) and HeLa cells (lane 4–6) separated by 12% SDS-PAGE. Blots were probed with antibodies specific to PUF60 and α-tubulin (top) or to U2AF65 and α-tubulin (middle). (B) Quantitation of PUF60 and U2AF65 protein levels. Blots were probed with a fluorescent secondary antibody and fluorescence was quantitated on a Fujifilm FLA-5100. The measurements showed a direct linear relationship between increasing amounts of input sample and fluorescence. Error bars represent the S.E.M of the three measurements from the blot shown in A. (0.40 MB TIF) [file pone.0000538.s007.tif]
