# Supplementary material for: Control of Pre-mRNA Splicing by the General Splicing Factors PUF60 and U2AF65
Source: PLoS One. 2007 Jun 20;2(6):e538. doi: 10.1371/journal.pone.0000538 (PMC1888729; doi:10.1371/journal.pone.0000538)
Supplement: Table S1 — Sequences of primers used in PCR reactions. (0.05 MB DOC) [file pone.0000538.s008.doc]

| **Primer** | **Sequence (5’ → 3’)** |
| --- | --- |
| **PUF60Hisstart** | CGAAGCTTGCCACCATGGTTCATCACCATCACCATCACATGGCGACGGCGACCATAGCTCTCC |
| **PUFresmutD** | CGGGATCCTCACGCAGAGAGGTCACTGTTATC |
| **U2AF35HISR** | CGAAGCTTGCCACCATGGTTCATCACCATCACCATCACATGGCGGAGTATCTGGCCTCCATC |
| **U2AF35STOPL** | CGGGATCCTCATCAGAATCGCCCAGATCTTTCAC |
| **PUF60NdeR** | GGCATATGGCGACGGCGACCATAG |
| **PUF60BamL** | CCGGATCCTCACGCAGAGAGGTCACTG |
| **PUF60resmutA** | CGAAGCTTGCCACCATGGCGACGGCGACCATAGCTC |
| **PUF60resmutB** | CTTGATGTTCCTGCCCCCCAGCATGACACTATTCATCTGTTCCAAGGCCAGCTGTGCAGCTTC* |
| **PUF60resmutC** | GAAGCTGCACAGCTGGCCTTGGAACAGATGAATAGTGTCATGCTGGGGGGCAGGAACATCAAG* |
| **PUF60resmutD** | CGGATCCTCACGCAGAGAGGTCACTGTTATC |
| **BamPUFstartR** | CGCGGATCCAGAATGGCGACGGCGACCATAGCTC |
| **XhoPUFstop** | CCGCTCGAGTCACGCAGAGAGAGGTCACTG |
| **forwardA** | GCAGAAATCATTGTCAAGATTTTTGTGGAGTTTTCCATAGCCTCTGAGAC* |
| **reverseA** | GAAAACTCCACAAAAATCTTGACAATGATTTCTGCATCCTCCTCCTCGCC* |
| **forwardB** | CGATCGggatccCCATGGCGACCATAGCTCTCCAGGTCAATGGCC |
| **reverseB** | CCATCGgaattcTCACGCAGAGAGGTCACTGTTATCAAAACGCTCCTGG |
| **APPexon6** | TGAAGACAAAGTAGTAGAAGTAG |
| **APPexon9** | CTGGGACATTCTCTCTCGGTGCTTG |
| **BINexon11** | CTGAGATCAGAGTGAACCATG |
| **BINexon15** | CACCCGCTCTGTAAAATTC |
| **MAPTexon9** | CAAGATCGGCTCCACTGAGAA |
| **MAPTexon13** | GGCGAGTCTACCATGTCGAT |
| **UBQLN1exon7** | CCTACATGAGAAGCATGATG |
| **UBQLN1exon9** | CTTCCGTTGCTAATGTCTG |
| **SMNexon6** | CGATCTCGAGATAATTCCCCCACCACCTCCC |
| **SMNexon8** | ATATGCGGCCGCCACATACGCCTCACATACA |

**Supplementary Table S1:** Sequences of primers used in PCR reactions.

* Underlined sequences indicate mutated positions.
